# Magnetic field-induced plasmonic enhancement of near infrared fluorescence from a Magnetoplasmonic nanoplatform for bioimaging applications

**DOI:** 10.1186/s12951-025-03691-6

**Published:** 2025-09-29

**Authors:** Siqi Gao, Jiantao Liu, Iuliia Golovynska, Zhenlong Huang, Yiqiang Wang, Hao Xie, Rana Zaki Abdul Bari, Hao Xu, Junle Qu, Tymish Y. Ohulchanskyy

**Affiliations:** Key Laboratory of Optoelectronic Devices and Systems of Ministry of Education and Guangdong Province, College of Physics and Optoelectronic Engineering, Shenzhen University, Shenzhen, 518060 China

**Keywords:** Magnetoplasmonic nanoparticles, Plasmon-enhanced fluorescence, Fluorescence lifetime imaging, Magnetic field-induced aggregation, Near infrared fluorescence bioimaging

## Abstract

A phenomenon of plasmon-enhanced fluorescence (PEF) arises from interactions between fluorophores and metal nanostructures, leading to a substantial amplification of the fluorescence signal. Herein, we report a magnetic field (MF) induced on-demand PEF from the magnetoplasmonic nanoplatform and demonstrate its application in near infrared (NIR) bioimaging. The developed magnetoplasmonic nanoparticles (~ 50 nm diameter) feature a core-shell-satellite architecture comprising a Fe_3_O_4_ magnetic core, a mesoporous silica (mSiO_2_) shell housing IR775-silane NIR dye, and surface-anchored gold (Au) seeds (satellites). Application of an external MF causes the magnetophoretic movement and aggregation of the nanoparticles (NPs), resulting in a formation of localized plasmonic hotspots and, consequently, in a plasmonic enhancement of NIR fluorescence from IR775 dye molecules. Correspondingly, a substantial reduction of the fluorescence lifetime in the MF-treated area was observed, in addition to the enhanced fluorescence intensity. In vivo studies with NPs subcutaneously injected into mice revealed MF-activated amplification of NiR fluorescence. At 6 h post-injection, the injected region treated by MF exhibited 2.1-fold stronger NIR fluorescence signal than the MF-untreated one; the fluorescence enhancement correlated with the reduction of the emission lifetime (from 0.68 ns to 0.47 ns). At 96 h post-injection, the MF-treated region exhibited 6.8-fold more intense NIR fluorescence. Histological analysis showed absence of toxicity from the injected NPs, revealing their biocompatibility. Hence, a considerable potential of MF-induced PEF with the magnetoplasmonic nanoplatform for targeted NIR fluorescence bioimaging was demonstrated. This work also introduces MF-induced PEF as a powerful strategy for spatiotemporal control of optical signals, offering new opportunities for targeted imaging and sensing.

**Graphical abstract:**

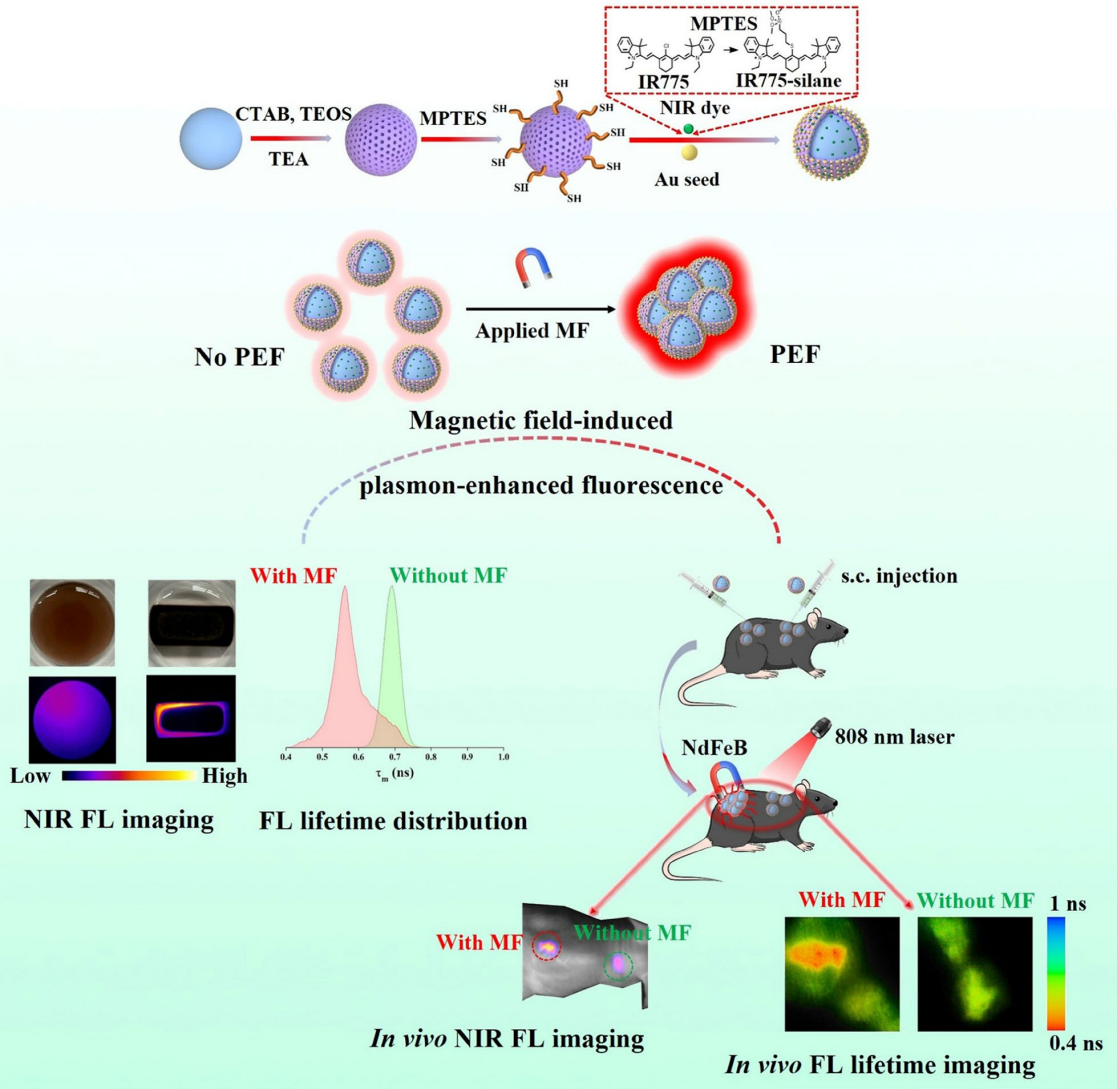

**Supplementary Information:**

The online version contains supplementary material available at 10.1186/s12951-025-03691-6.

## Introduction

Plasmon-enhanced fluorescence (PEF) is a plasmonic phenomenon first reported in 1980 [1]. It originates from the plasmon resonance coupling between the frequencies emitted by the fluorophore and local surface plasmon resonance (LSPR) of the metal particles (NPs) that can result in a significant enhancement in the emission intensity [2, 3]. Use of PEF phenomenon can be advantageous for fluorescence bioimaging, particularly for near infrared (NIR) fluorescence imaging in vivo, as it would specifically benefit from an enhancement of fluorescence from imaging probes, allowing for deeper tissue imaging with a low background [4–6]. However, only a few studies reported PEF nanoformulations in in vivo imaging systems; PEF nanoformulations designed for in vivo fluorescence imaging generally lack high specificity/targeting ability and may reveal some toxicity in in vivo applications; At the same time, the PEF effect in nanoformulations renders to be unstable under the in vivo fluorescence imaging conditions [7–9]. Thus, to be employed in NIR imaging in vivo, PEF nanoformulations should possess good biocompatibility along with targeting ability for a specific application (e.g., tumor targeting in cancer theranostics).

The PEF nanoformulations used in biosensing applications usually rely on the use of specific antibodies or aptamers as molecular recognition elements, which allow for specific binding to target analytes through the antibody-antigen or aptamer-nucleic acid interactions [10, 11]. However, when PEF nanoformulations come into contact with biological fluids in vivo, they encounter thousands of proteins, which reduces the detection sensitivity and specificity owing to inevitable non-specific adsorption [12], largely limiting an application of this approach for the in vivo NIR fluorescence imaging. On the other hand, an application of magnetic field (MF) targeting for localized bioimaging and therapy is widely reported, utilizing MF to magneticphoretically attract NPs loaded with imaging and/or therapeutic agents to targeted areas, where MF is the strongest. Unlike molecular targeting, magnetic targeting based on physical interactions is not limited by the specific receptor expression and maybe a more general active-targeting approach [13–17]. Recently, Y. Liu and colleagues proposed a multifunctional nanoplatform of upconversion/iron oxide (UCNP/Fe_3_O_4_) NPs for magnetically targeted NIR-II imaging. The NIR-II imaging in vivo uncovers that UCNP/Fe_3_O_4_ NPs tend to migrate toward the tumor under influence of MF from a magnet placed near the tumor, and exhibit intense tumor accumulation, about 6-fold higher than that without magnetic targeting [18]. D. Ni with colleagues reported magnetic NPs with ^89^Zr radiolabeling and porphyrin molecules (^89^Zr-MNP/TCPP) that exhibited a high tumor accumulation and significantly enhanced the fluorescence intensity under the presence of an external MF [19]. In addition, L. Chen with colleagues reported magnetoplasmonic nanocomposites of Au-shelled upconversion/iron oxide (MFNPs), which showed an ability to be magnetophoretically controlled and concentrated using the external MF. With the help of MF, the fluorescence intensity of tumor position was about 8-fold higher than that without MF targeting [20]. Hence, the application of an external MF is considered a simple but efficient method that can target some specific locations for in vivo fluorescence imaging.

Very recently, we have reported Fe_3_O_4_@mSiO_2_@Au core@shell@satellites magnetoplasmonic NPs loaded with the chemotherapeutic drug doxorubicin for a magnetic field-induced and targeted combination of near-infrared photothermal therapy (NIR PTT) and chemotherapy [21]. When an external MF is applied to the dispersion of these NPs, it results in the magnetophoretic movement and aggregation of the NPs. The MF-induced aggregates reveal a notable absorption in NiR spectral range due to the plasmon resonance coupling between the Au satellites. As a result, an enhanced photothermal effect is observed in MF-treated NPs dispersion under 808 nm laser irradiation. The MF-induced, tumor targeted combination of NiR PTT with DOX chemotherapeutic action effectively kills cancer cells in vitro and restricts tumor growth in 4T1-tumor-bearing mice in vivo. Hence, our study revealed strong enhancement of NIR absorption of the magnetopasmonic NPs, which appeared only under the external magnetic field, resulting from the aggregation-induced plasmon resonance coupling. In this regard, it would be naturally to suggest that a presence of fluorophores within MF-induced aggregates may lead to an appearance of MF-induced PEF. This study reports a PEF effect generated by magnetoplasmonic nanoparticles (Fe_3_O_4_@mSiO_2_@Au-IR775 NPs) under applied MF, which significantly amplifies fluorescence emission, while avoiding the aggregation-induced quenching (ACQ) effect that typically occurs in conventional fluorescent systems. It is important to emphasize that use of external magnetic field to induce-dynamic PEF for bioimaging application has not been reported before, according to our knowledge. The proposed approach can be of interest for many bioimaging applications [e.g., it allows for on-demand localized highlighting of the region of interest (ROI)]. On the other hand, the PEF response to MF can be calibrated to probe MF strength at ROI. Furthermore, it should be noted that only a few studies have utilized PEF systems in in vivo analytical applications. When PEF probes come into contact with biological fluids in situ, they encounter several thousand proteins, which reduces the detection sensitivity and specificity owing to inevitable non-specific adsorption. In this regard, the MF-induced PEF effect reported in this study can simultaneously addresses two key limitations of conventional PEF probes in in vivo applications: (i) overcoming the lack of target specificity through magnetic targeting, and (ii) enabling PEF on-demand at desired sites via MF application.

**Scheme 1:**
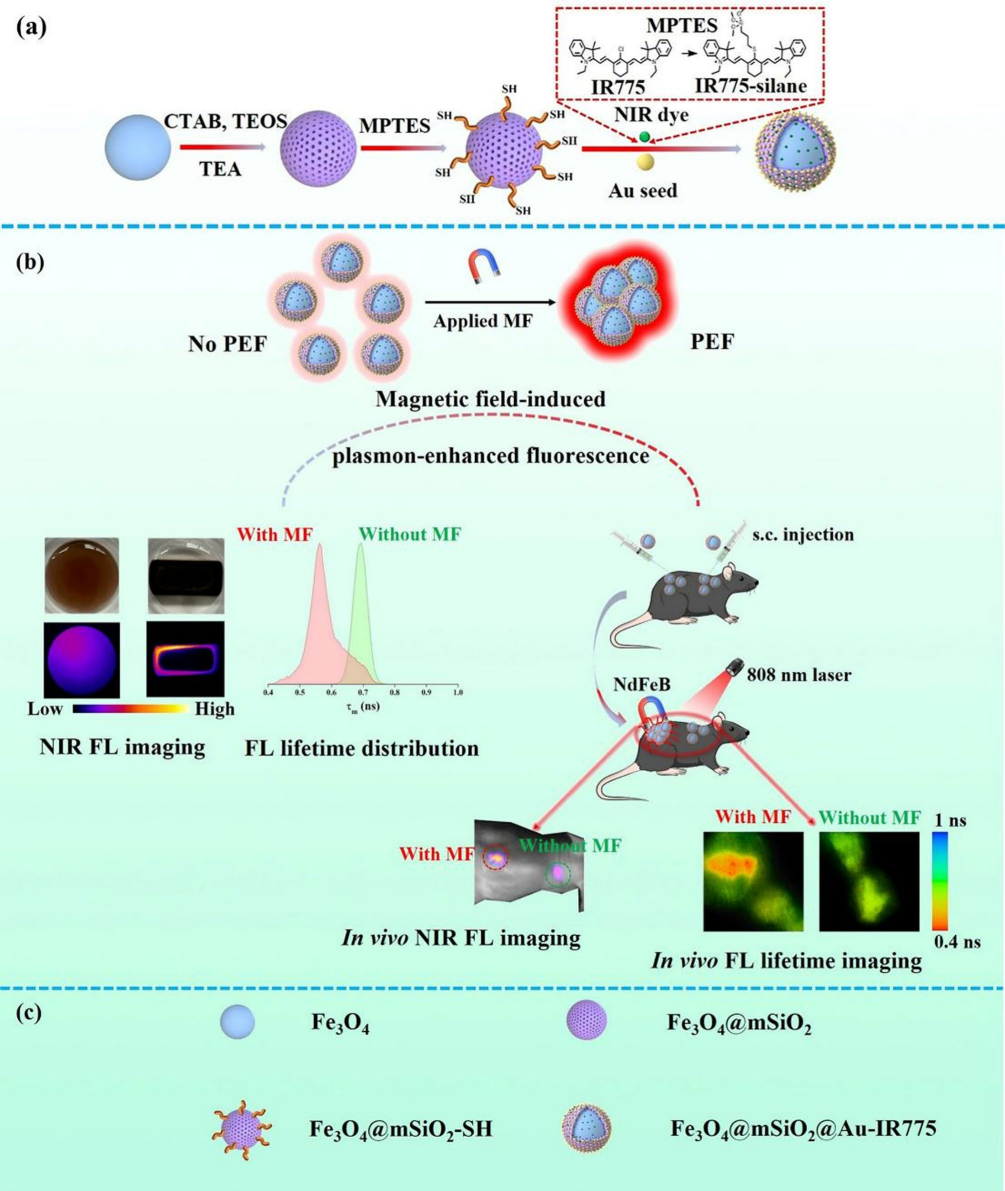
Schematic illustration of (**a**) synthesis of magnetoplasmonic Fe_3_O_4_@mSiO_2_@Au-IR775 nanoparticles, (**b**) plasmon-enhanced NIR fluorescence induced in situ by magnetic field locally applied to magnetoplasmonic NPs, and (**c**) depictions of the nanoparticles used in (**a**)

Herein, we report a MF-induced PEF effect using Fe_3_O_4_@mSiO_2_@Au magnetoplasmonic nanoparticles surface-conjugated with a functionalized NIR fluorescent dye, IR775-silane (Scheme 1). The application of an external MF was shown to lead to the formation of Fe_3_O_4_@mSiO_2_@Au IR775 aggregates causing an increase in the IR775 fluorescence intensity with a simultaneous decrease in the fluorescence lifetime, which was attributed to the PEF effect resulted from the aggregation-induced increase in the amount of Au satellites in proximity to the IR775 fluorophores. In the in vivo studies, we achieved an efficient, magnetically induced PEF effect in the injected Fe_3_O_4_@mSiO_2_@Au-IR775 NPs, as the NiR fluorescence was significantly enhanced by the application of external MF. It also led to the prolonged accumulation of the NPs in the targeted region. 90 h after ending of MF application, the fluorescence signal from the site of the injection of Fe_3_O_4_@mSiO_2_@Au-IR775 NPs, which was treated by MF for 6 h, was much higher than that from the injection site where MF was not applied. Moreover, NIR fluorescence lifetime imaging in vivo further confirmed the MF-induced PEF effect in the injected Fe_3_O_4_@mSiO_2_@Au-IR775 NPs: after the MF application, the NIR fluorescence lifetime significantly decreased in comparison with that before MF was applied, while the lifetime of the fluorescence from the control, MF-untreated site of Fe_3_O_4_@mSiO_2_@Au-IR775 NPs injection remained the same. Taking into account the results of the histological studies revealing absence of noticeable toxicity from the injected NPs, this work provides a feasible but effective approach to induce PEF effect for in vivo NIR fluorescence imaging. At the same time, a possibility to induce and control PEF with the locally applied MF can provide interesting opportunities for multiple imaging and sensing applications.

## Materials and methods

### Materials

1-octadecene (ODE), dibenzyl ether, 1-tetradecene, iron (III) acetylacetonate (Fe(acac)_3_), oleic acid (OA), anhydrous ethanol, hexane, chloroform, cetyltrimethylammonium bromide (CTAB), triethanolamine (TEA), tetraethyl orthosilicate (TEOS), (3-mercaptopropyl) trimethoxysilane (MPTES), ammonium nitrate, dimethylformamide (DMF), 2-[2-[2-chloro-3-[2-(1,3,3-trimethylindol-1-ium-2-yl)ethenyl]cyclohex-2-en-1-ylidene]ethylidene]−1,3,3-trimethylindole chloride (IR775), chloroauric acid (HAuCl_4_), sodium citrate, and sodium borohydride (NaBH_4_) were purchased from Sinopharm Chemical Reagent Co (China). All reagents were directly used without purification. A rectangular (20 × 10 × 4 mm) and a round (5 mm diameter, 1 mm thick) NdFeB magnets were purchased from Taobao and used for in vitro and in vivo experiments, respectively.

### Preparation of Fe3O4

In brief, a mixture of ODE (50 mL), dibenzyl ether (50 mL), and 1-tetradecene (15 mL) was used as a high-boiling solvent. Then, Fe(acac)_3_ (5 mmol), and OA (20 mmol) were added in the mixed solvent. This resulting mixture was heated to 70℃ under a vacuum and kept at this temperature for 1 h with vigorous magnetic stirring. Subsequently, the temperature of the mixture was increased to 290 °C under a constant flow of argon gas and kept at this temperature for another 1 h. Finally, the mixture was cooled to room temperature under a constant flow of argon gas. The purification of the iron oxide was performed by adding anhydrous ethanol, the black mixture was centrifugation at 12,000 rpm for 20 min, and then the product was washed several times with a mixture of anhydrous ethanol and hexane to remove the residual reactants. Finally, iron oxide was dissolved in chloroform and stored in a refrigerator at 4 °C.

### Preparation of Fe_3_O_4_@mSiO_2_

Firstly, CTAB (0.4 g) and TEA (0.06 g) were dissolved in deionized water (20 mL) under magnetic stirring, and then iron oxide (2 mg, in 1 mL chloroform) was added to the above solution, the mixed solution was continuously sonicated for 30 min. The chloroform in the system was removed by a rotary evaporator (80 °C). After these injections, the resulting solution was kept at 80 °C for 1 h under magnetic stirring, and the TEOS (100 µL) was added dropwise into the above solution. The reaction system was stirred for 4 h in the thermostatic water bath at 80 °C. The product was collected by centrifugation (12000 rpm, 10 min) and washed several times with ethanol. To remove the surfactants (CTAB), the Fe_3_O_4_@mSiO_2_ solution was obtained by stirring in a solution of ammonium nitrate in ethanol (1 wt%, 3 h) for two rounds.

### Preparation of MPTES-modified Fe_3_O_4_@mSiO_2_

Fe_3_O_4_@mSiO_2_ nanoparticles (1 mg) were dispersed in ethanol by ultrasonication for 30 min. Next, MPTES (100 µL, 10% in ethanol) was added to the Fe_3_O_4_@mSiO_2_ solution and sonication continued for another 20 min. The resulting solution was placed in the shaker at room temperature for two days. Then, the obtained Fe_3_O_4_@mSiO_2_-SH nanoparticles were washed three times with ethanol to remove the unbound organosilane reagents and redispersed into 10 mL ethanol.

### Preparation of IR775-silane precursor

IR775-conjugated silane precursor was prepared as depicted in Figure S1. In a typical procedure, NIR fluorescent dye IR775 (30 mg, 0.059 mmol) was dissolved in anhydrous DMF (1 mL). To this, TEA (45 µL, 0.3 mmol) and MPTES (60 µL, 0.3 mmol) was added, and the resultant mixture was stirred for 12 h at 55℃ under N_2_ atmosphere to evaporate the organic solvent. The final obtained product was directly used to load into the mesoporous silica shell.

### Preparation of Fe_3_O_4_@mSiO_2_@Au-IR775

20 mL solution of HAuCl_4_ (0.25 mM) and sodium citrate (0.25 mM) were mixed under vigorous magnetic stirring. Then, 0.6 mL of freshly prepared 100 mM NaBH_4_ solution was added to the above solution. The formation of Au NPs was confirmed by observing the change in its color from pink to Light red. Finally, the resulting mixture was continuously stirred for 30 min at room temperature. 2 mL of the prepared Fe_3_O_4_@mSiO_2_-SH solution was mixed with IR775-silane methanol solution at different concentrations (5 µg/mL, 9 µg/mL, and 12.5 µg/mL) for two days. Then, the resulting solution was added dropwise into 4 mL of fresh Au nanoparticle solution, and the mixed solution was continuously sonicated for 30 min. Then, the reaction was carried out with vortex stirring for another one day. Finally, the obtained product was washed with water three times and redispersed in the deionized water.

### Characterization

The absorption spectra were measured with a UV-Vis spectrophotometer LAMBDA 750 (PerkinElmer, USA). Emission spectra were recorded using a Fluorolog-3 spectrofluorometer (Horiba, Japan). DLS and Zeta potential were performed using a Nano-ZS90 Zetasizer (Malvern Panalytical, United Kingdom). Transmission electron microscopy (TEM) was performed using a JEM2100PLUS TEM instrument (JEOL, Japan). Elemental mapping analyses was performed using a JEM-F200 (HRP) instrument. Magnetization measurements were performed using a LakeShore7404 instrument. A commercial fluorescence lifetime imaging system (DCS-120, Becker & Hickl, GmbH, Germany) equipped with a confocal microscope was utilized to perform fluorescence lifetime imaging analysis. The NiR fluorescence imaging was performed using a homemade NiR imaging system with 850 nm long-pass (LP) filter at 808 nm laser irradiation.

### FDTD simulation

The FDTD simulations were conducted using Lumerical FDTD solutions 2020 software, simulation mode of Fe_3_O_4_@mSiO_2_@Au nanostructures was built with 3D MAX, and the results analyses were processing with Matlab 2022. In FDTD simulation, Palik was chosen as SiO_2_ and Au materials, simulation time was setting as 1000 fs, simulation temperature was 300 K, boundary condition was perfectly matched layer (PML). Override mesh was added on Fe_3_O_4_@mSiO_2_@Au nanostructures, maximum mesh step and minimum mesh step of X and Y were setting as 5 nm and 0.25 nm, respectively. The wavelength of incident source is 808 nm. Frequency-domain field profile monitor was added to visualize electric field information. After simulation, data were export into Matlab via FDTD scripts, and further analyses were processed in MATLAB 2022.

### Fluorescence lifetime imaging microscopy (FLIM)

Fluorescence lifetime imaging microscopy (FLIM) was conducted using a multi-modal fluorescence microscope (SKMM-1, KAYJA-OPTICS). A femtosecond laser (Chameleon, Coherent) was set to 808 nm. To employ FLIM for characterization of NIR fluorescence from Fe_3_O_4_@mSiO_2_@Au-IR775 NPs with and without applied MF, NPs aqueous dispersion was placed in Petri dishes. To introduce a magnetic field, a rectangular NdFeB magnet (20 mm × 10 mm × 4 mm) was placed under the dish bottom. A 2.5× objective lens was installed in the microscope to focus the excitation light onto the sample and collect fluorescence signal. The fluorescence lifetime images were acquired and stored by the controlling software SPCM64 (version 9.76, Becker & Hickl, GmbH, Germany). The software used for FLIM analysis was SPCImage 8.4. The pixel dwell time was set to 3.2 µs, and the acquisition time for a Lifetime image consisting of 512× 512 pixels in a single scan was ~ 40 s.

### NIR fluorescence imaging

To perform a NIR fluorescence imaging of Fe_3_O_4_@mSiO_2_@Au-IR775 NPs with and without applied MF, Fe_3_O_4_@mSiO_2_@Au-IR775 aqueous dispersion (500 µL, 1 mg/mL, IR775 concentration = 9 µg/mL) was placed in Petri dishes. To introduce a magnetic field, a rectangular NdFeB magnet (20 × 10 × 4 mm) was placed the dishes bottom. The NIR fluorescence images of Fe_3_O_4_@mSiO_2_@Au-IR775 NPs before and after applied MF were captured using a homemade NiR imaging system based on InGaAs SWIR imaging camera and 808 nm laser excitation (power density = 20 mW/cm^2^, exposure time = 20 ms-200 ms).

All animal experiments studies were performed in compliance with the guidelines established by the Animal Ethical and Welfare Committee of Shenzhen University, which adhere to international standards for animal care (Approval No. SZUHSC-01). Female nude mice (4–6 weeks old) were used for in vivo studies. For in vivo imaging, Fe_3_O_4_@mSiO_2_@Au-IR775 nanoformulation (200 µL, 1 mg/mL, IR775 concentration = 9 µg/mL) was subcutaneously injected into the different regions on the back of the mouse. Then, the corresponding NiR fluorescence images of different injection regions from mouse were immediately obtained at 0 h. To introduce a magnetic field, a small round NdFeB magnet (5 mm diameter, 1 mm thick) was placed the one injection region on the back of mouse, and without any treatment for another injection region. At 6 h, 24 h, 36 h, 48 h, 72 h, and 96 h post-injection, the NiR fluorescence images of the mice were captured using a homemade NiR imaging system based on InGaAs SWIR imaging camera and 808 nm laser excitation (power density = 160 mW/cm^2^, exposure time = 200 ms). After imaging, the mice were sacrificed and major organs (heart, liver, spleen, lungs, and kidneys) and skin were harvested for ex vivo NIR fluorescence imaging.

### Histopathological analysis

For histological examination of lungs, liver, spleen, kidneys, and heart a standard formalin-fixed paraffin-embedding method was used. Serial slices (4 μm thickness, 10 slices per organ) were obtained and stained with hematoxylin and eosin (H&E) according to the standard method. Representative histological images were obtained using an inverted Nikon fluorescence microscope and analyzed with ImageJ software. The severity of lungs, liver, spleen, kidneys, and heart injury was determined using a histological index of quantitative assessment (IQA). Briefly, 6 slices were randomly selected from each group of mice, and 3 fields of each section were reviewed under a microscope. The scoring was determined by scale from 0 to 3 based on the degree of pathological changes for each organ: absence of pathology (0) presence of mild pathological changes (1), moderate pathological changes (2), or severe pathological changes (3).

### Statistical analysis

All data were presented as the mean standard deviation (SD) from at least three independent experiments (*n* ≥ 3). The statistical differences in the data were determined using a One-Sample t-Test analysis within Origin 9_64 software. *p < 0.05, **p < 0.01, and ***p < 0.001 were considered statistically significant.

## Results and discussion

**Fig. 1:**
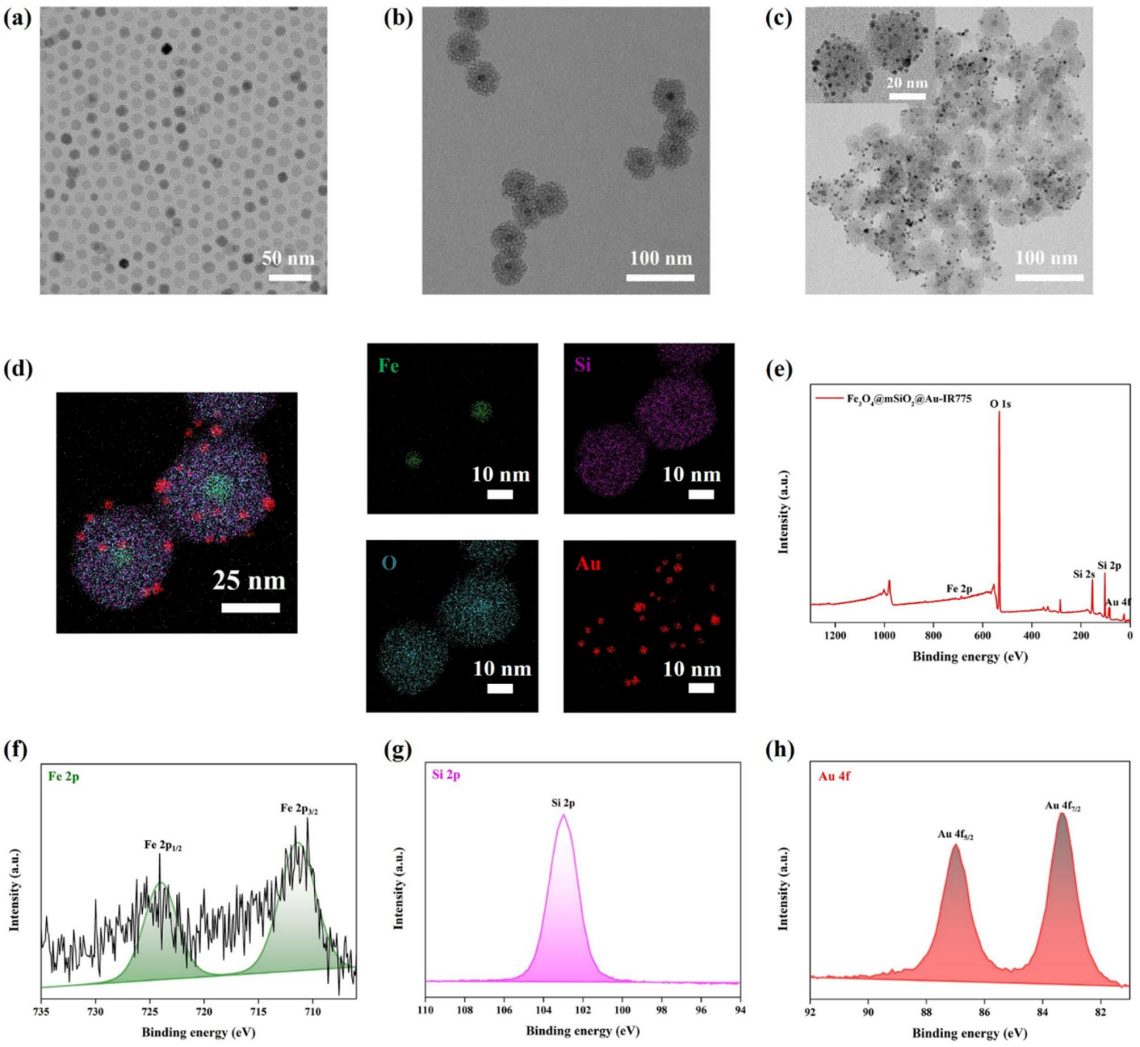
TEM images of Fe_3_O_4_ NPs (**a**), Fe_3_O_4_@mSiO_2_ NPs (**b**), Fe_3_O_4_@mSiO_2_@Au-IR775 NPs (**c**). (**d**) Elemental mappings showing the spatial distribution of Fe (green), Si (magenta), O (cyan), and Au (red) in the Fe_3_O_4_@mSiO_2_@Au-IR775 NPs. (**e**) XPS spectra of the Fe_3_O_4_@mSiO_2_@Au-IR775 NPs, and (**f**) Fe 2p, (**g**) Si 2p and (**h**) Au 4f peaks from Fe_3_O_4_@mSiO_2_@Au-IR775 XPS spectra shown in (**e**)

The Fe_3_O_4_@mSiO_2_@Au-IR775 core@shell@satellites NPs were prepared by a layer-by-layer assembly approach using iron oxide, mesoporous silica, and gold as building blocks. Monodisperse Fe_3_O_4_ NPs with an average diameter of 8 nm (Fig. 1(a)) were first synthesized using a modified thermal decomposition protocol [22]. Then, a mSiO_2_ shell (∼18 nm thick) was subsequently coated on the Fe_3_O_4_ core using a reverse microemulsion approach [23]. A TEM performed after removal of the surfactant CTAB confirmed the formation of well-defined Fe_3_O_4_@mSiO_2_ NPs with a 44 nm average diameter, single-core structure and a uniform mSiO_2_ shell with ∼18 nm thickness (Fig. 1(b)). Next, MPTES was used as a silane coupling reagent, which provided negatively charged sulfhydryl groups on the silica surface, allowing for attachment of the pre-synthesized Au NPs through formation of a strong Au-S bond. Finally, the functionalized NIR fluorescent dye IR775-silane was loaded into the mesoporous structure via a post-synthetic grafting method (Figure S1 and Experimental Section in Supporting Information); this process was facilitated by the abundant surface silanol groups (Si-OH) of mSiO_2_ that serve as effective anchoring sites [24]. In such a way, Fe_3_O_4_@mSiO_2_@Au-IR775 NPs with core@shell@satellites structure were successfully obtained (Fig. 1(c)). The element mapping images of Fe_3_O_4_@mSiO_2_@Au-IR775 NPs (Fig. 1(d)) prove that the prepared NPs have core of Fe (core), the mSiO_2_ shell in the middle layer and the outer Au satellites. In order to further verify the structure of Fe_3_O_4_@mSiO_2_@Au-IR775 nanocomposites, energy dispersive spectroscopy (EDS) analysis was performed, quantitatively verifying the presence of Fe, Si, O, and Au elements (Figure S2 and Table S1); this result is in good agreement with TEM findings. In addition, X-ray photoelectron spectroscopy (XPS) was performed to further examine the chemical composition of Fe_3_O_4_@mSiO_2_@Au-IR775 NPs. Figure 1(e) shows the XPS spectrum of Fe_3_O_4_@mSiO_2_@Au-IR775 NPs over the scan range 0-1300 eV. As shown in Fig. 1(f), after the Fe_3_O_4_ core is coated with a mSiO_2_ shell, the Fe 2p peak signal becomes very weak due to the shallow XPS detection depth Limit of 10 nm [25], and the curve fitting analysis clearly reveals the characteristic peaks corresponding to Fe 2p_1/2_ (724.0 eV) and Fe 2p_3/2_ (711.4 eV). A clear Si 2p peak (103.0 eV) is observed, confirming a presence of Si element in the mSiO_2_ shell (Fig. 1(g)). The presence of Au 4f_7/2_ (83.4 eV) and Au 4f_5/2_ (87.0 eV) peaks further verified that the Fe_3_O_4_@mSiO_2_ NPs were decorated with Au satellites (Fig. 1(h)). Based on the TEM, EDX, and XPS results, we concluded that the Fe_3_O_4_@mSiO_2_@Au-IR775 NPs with the core@shell@satellites structure were successfully synthesized.

**Fig. 2:**
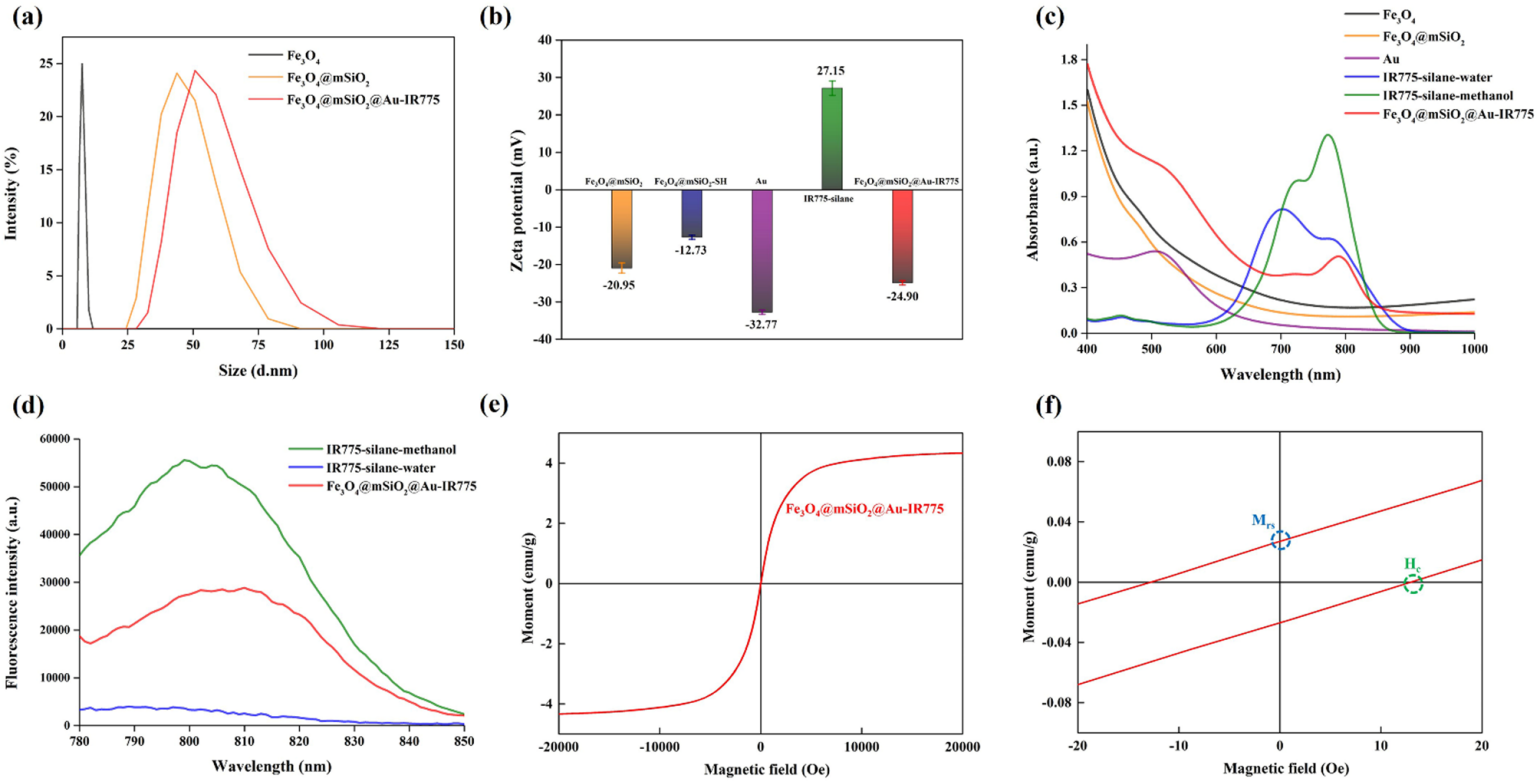
(**a**) DLS data for Fe_3_O_4_, Fe_3_O_4_@mSiO_2_ and Fe_3_O_4_@mSiO_2_@Au-IR775 NPs. (**b**) ζ potentials for Fe_3_O_4_@mSiO_2_, MPTES-modified Fe_3_O_4_@mSiO_2_, Au, IR775-silane in water, and Fe_3_O_4_@mSiO_2_@Au-IR775 NPs. (**c**) Absorbance of Fe_3_O_4_, Fe_3_O_4_@mSiO_2_, Au, IR775-silane in water, IR775-silane in methanol, and Fe_3_O_4_@mSiO_2_@Au-IR775 NPs. (**d**) Spectral of fluorescence intensity of IR775-silane in water, IR775-silane in methanol, and Fe_3_O_4_@mSiO_2_@Au-IR775 NPs in water. (**e**) Hysteresis loop of the magnetophoretic response of the Fe_3_O_4_@mSiO_2_@Au-IR775 NPs, and (**f**) the curve of Fe_3_O_4_@mSiO_2_@Au-IR775 NPs with the external magnetic field is near zero, wherein the two markers present the values of saturation remanence (M_rs_) and coercivity (H_c_), respectively

Figure 2(a) and Figure S3 shows the dynamic light scattering (DLS) results revealing that the hydrodynamic diameters of Fe_3_O_4_, Fe_3_O_4_@mSiO_2_ and Fe_3_O_4_@mSiO_2_@Au-IR775 NPs are 7.6 nm, 46.7 nm and 55.9 nm, respectively. The progressive increase in nanoparticle size provides indirect evidence for the successful coating of Fe_3_O_4_ core with the mSiO_2_ shell and subsequent conjugation of Au seeds (Table S2). The TEM and DLS results for pre-synthesized Au NPs are shown in Figure S4. Furthermore, the ζ potential of the Fe_3_O_4_@mSiO_2_ NPs was found to change from − 20.95 mV to −12.73 mV as a result of modification with MPTES; the following change of ζ potential from − 12.73 mV to −24.90 mV is associated with the Au NPs (−32.77 mV) and IR775-silane (27.15 mV) (Fig. 2(b)). Interestingly, zeta potential of Fe_3_O_4_@mSiO_2_@Au-IR775 NPs is not only different from that of Fe_3_O_4_@mSiO_2_-SH (confirming conjugation of Au NPs), it is also more negative (−24.90 mV for Fe_3_O_4_@mSiO_2_@Au-IR775 NPs vs. + 27.15 mV for IR775-silane dye). As IR775 dye is positively charged, change of Fe_3_O_4_@mSiO_2_@Au-IR775 zeta potential to more negative in comparison with IR775-silane dye suggests that IR775-silane dye is loaded inside mesopore channel through Fe_3_O_4_@mSiO_2_@Au-IR775 NPs but not stick to the surface of Au NPs. Overall, these characterization results on core-shell-satellites NPs are similar to those reported by us [21]. The optical properties of the NPs were investigated using optical absorption and fluorescence spectroscopies (Fig. 2(c, d)). While Fe_3_O_4_ and Fe_3_O_4_@mSiO_2_ NPs exhibited no distinct absorption bands in 400–1000 nm spectral range, Fe_3_O_4_@mSiO_2_@Au-IR775 NPs displayed clear absorption bands peaked at ~ 520 nm and ~ 787 nm, which correspond to the localized surface plasmon resonance (LSPR) of Au satellites and the characteristic absorption of IR775-silane within the mSiO_2_ shell, respectively. Notably, the IR775-silane absorption band in the Fe_3_O_4_@mSiO_2_@Au NPs exhibited a slight red-shift (787 nm) compared to free IR775-silane in methanol (775 nm), revealing change in polarity of the environment for the IR775-silane molecules [26–28]. A similar red-shift was observed in the fluorescence spectra, where the emission peak of IR775-silane shifted from 800 nm (in methanol) to 810 nm in the Fe_3_O_4_@mSiO_2_@Au-IR775 aqueous dispersion (Fig. 2(d)). It is worth noting that the fluorescence intensity of IR775-silane in water was significantly quenched relatively to its methanol solution and the Fe_3_O_4_@mSiO_2_@Au NP-loaded dispersion. This attenuation likely arises from the propensity of IR775-silane to aggregate in aqueous media, leading to aggregation-caused quenching (Figure S5) [29]. This aggregation is confirmed by the appearance of the pronounced absorption band at ~ 710 nm; such a blue-shifted absorption band is knowingly associated with H-aggregates (e.g., dimers) of dye molecules [30]. Furthermore, it was found that the absorption spectrum of Fe_3_O_4_@mSiO_2_@Au-IR775 NPs dispersion noticeably changed when MF was applied, with the most significant change (i.e., appearance of the intense absorption without a pronounced peak) in the NIR spectral range [21]. In addition, a clear red-shift of visible absorption from 520 nm to 600 nm also indicated the transformation of monodispersed Au NPs to formed “dimer” and “trimer” structures of Au NPs where strong plasmon coupling effects exist (Figure S6(a)) [31, 32], resulting in a significant enhancement of the fluorescence signal of Fe_3_O_4_@mSiO_2_@Au-IR775 NPs after the application of a MF (Figure S6(b)). It should be noted that similar changes were observed in spectra of Fe_3_O_4_@mSiO_2_@Au-DOX NPs reported by us recently. Specifically, an appearance of NIR absorption caused by MF-induced plasmonic coupling of these core@shell@satellites NPs allowed us to achieve MF-induced NiR PTT and, sequentially, introduce the MF-induced, tumor targeted combination of NiR PTT with chemotherapeutic action, which allows for an efficient kill of cancer cells in vitro and restriction of a tumor growth in 4T1-tumor-bearing mice in vivo [21].

As can be seen in Figures. S7 and S8, the fluorescence of IR775-silane in core-shell-satellites NPs reached saturation at loading concentration of 9 µg/mL, pointing towards aggregation of the dye fluorophores within the NPs at this and higher concentrations, which causes the fluorescence quenching [33, 34]. The colloidal stability of Fe_3_O_4_@mSiO_2_@Au-IR775 NPs under physiological conditions was evaluated using DLS technique, for NPs dispersed in deionized water (DI), PBS (pH = 7.4), and DMEM. As it is seen in Figure S9, though an initial increase of the hydrodynamic diameter of NPs in PBS and DMEM (in comparison with DI) has been observed (which is evidently associated with a formation of a solvation shell in PBS and DMEM), no significant changes in size of Fe_3_O_4_@mSiO_2_@Au-IR775 NPs were found after 3 days of storage. Furthermore, the colloidal stability of Fe_3_O_4_@mSiO_2_@Au-IR775 NPs, we have performed zeta potential measurements of Fe_3_O_4_@mSiO_2_@Au-IR775 NPs under different pH conditions. No significant changes in zeta potential of Fe_3_O_4_@mSiO_2_@Au-IR775 NPs were observed under different pH conditions (Figure S10). The fluorescence spectra of Fe_3_O_4_@mSiO_2_@Au-IR775 NPs at various pH (Figure S11) are found to be quite similar; a slightly more instence fluorescence of Fe_3_O_4_@mSiO_2_@Au-IR775 NPs at pH = 8 might be an artefact. We believe that these results further imply that the Fe_3_O_4_@mSiO_2_@Au-IR775 NPs are fairly stable at various pH. In addition, the photostability of Fe_3_O_4_@mSiO_2_@Au-IR775 NPs with and without applied MF was investigated. When aqueous dispersions of Fe_3_O_4_@mSiO_2_@Au-IR775 NPs (with various pH) were irradiated with an 808 nm laser (power density = 160 mW/cm^2^) for 15 min, the fluorescence was noticeable reduced. The changes were less noticeable for the fluorescence from the MF-induced aggregates of Fe_3_O_4_@mSiO_2_@Au-IR775 NPs (Figure S12).

Figure 2(e) shows the magnetization characterization of Fe_3_O_4_@mSiO_2_@Au-IR775 at room temperature. One can see that the saturation magnetization (Ms) of Fe_3_O_4_@mSiO_2_@Au-IR775 is about 4.33 emu/g. Furthermore, Fig. 2(f) shows the magnetization plot for near the zero magnetic field. Saturation remanence (Mrs) and coercivity (Hc) can be determined from the intersection of the hysteresis loop with two axes at 0.027 emu/g and 12.92 Oe, respectively. These two values indicate that a rather low residual magnetization is present when the external magnetic field is removed and that a low-intensity magnetic field is required to reduce the magnetization to zero. These results reveal that the Fe_3_O_4_ in Fe_3_O_4_@mSiO_2_@Au-IR775 is in the superparamagnetic state [35, 36].

**Fig. 3:**
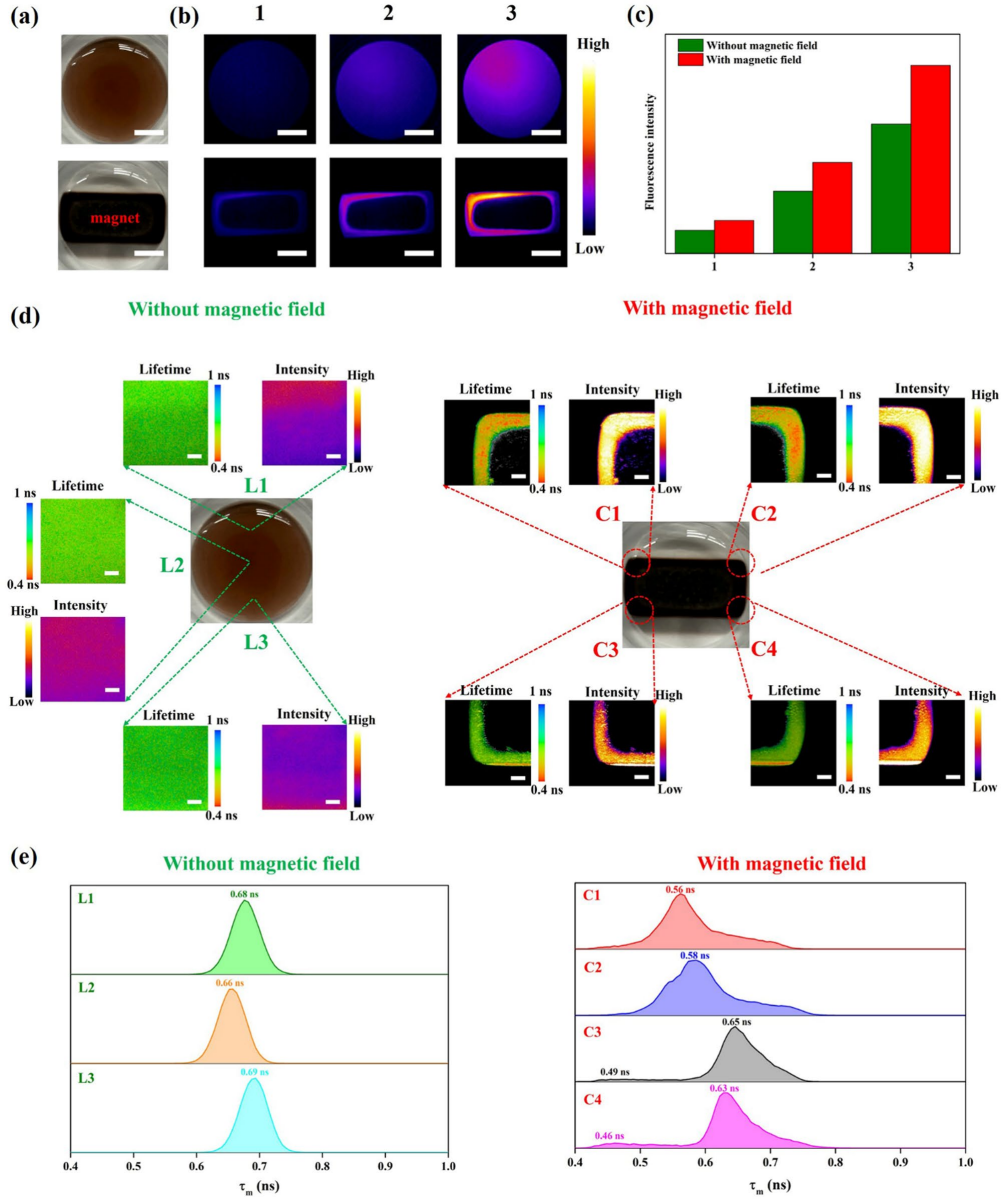
(**a**) Photographs of Fe_3_O_4_@mSiO_2_@Au-IR775 aqueous dispersion in Petri dish without (upper row) and with (down row) applied MF, and (**b**) the corresponding NIR fluorescence images obtained by imaging camera at different exposure times (1: 20 ms, 2: 50 ms, and 3: 100 ms). Scale bar is 5 mm. (**c**) Fluorescence intensities for different camera exposure times using 808 nm laser irradiation. (**d**) Average fluorescence lifetime (τ_m_) images and (**e**) lifetime distribution of Fe_3_O_4_@mSiO_2_@Au-IR775 aqueous dispersions without and with applied MF. Scale bar is 1 mm

At the next stage, a behavior of NIR fluorescence from Fe_3_O_4_@mSiO_2_@Au-IR775 NPs under MF and without it was explored. While Fig. 3(a) shows photographic images of Fe_3_O_4_@mSiO_2_@Au-IR775 NPs water dispersion in a Petri dish without and with magnet application, Fig. 3(b) presents corresponding NIR fluorescence images at different imaging camera exposure times (20, 50, and 100 ms). Notably, the Fe_3_O_4_@mSiO_2_@Au-IR775 at the corner of magnet (where MF is the strongest) produced the much stronger fluorescence compared to other areas under MF application, which can be associated with a plasmon coupling effect produced by the Au NPs becoming adjacent due to an MF-induced formation of Fe_3_O_4_@mSiO_2_@Au-IR775 aggregates/clusters [21, 37, 38]. As visualized in Fig. 3(c), the total NIR fluorescence signal from the images of NPs under MF is clearly higher than that from NPs in absence of MF. It is worth noting that the NIR fluorescence from MF-gathered Fe_3_O_4_@mSiO_2_@Au-IR775 remains stable even 5 days after MF application (Figure S13). It should be noted that superparamagnetic NPs are well-established to aggregate under an external MF. Particularly, when exposed to MF, each nanoparticle acquires a magnetic dipole moment, leading to mutual attraction through magnetic dipole-dipole interactions. This phenomenon effectively drives the NPs together to form aggregates, a process commonly referred to as “magnetic chaining” [39]. Figure S14 shows TEM image illustrating the formation of Fe_3_O_4_@mSiO_2_@Au-IR775 NPs aggregates. In turn, the magnetically-activated formation of the Fe_3_O_4_@mSiO_2_@Au aggregates leads to a shortening of distance between the satellite Au NPs, which, in turn, can cause their surface plasmon resonance coupling [21]. As illustrated in Figures S15-S19, the finite-difference time domain (FDTD) simulations reveal that the MF-induced formation of “dimer” and “trimer” structures of Fe_3_O_4_@mSiO_2_@Au NPs leads to an appearance of intense “hot spots” in electric field strength when the interparticle distance between adjacent Au NPs reduces. When the distance between adjacent Au NPs in “dimer” and “trimer” structures of Fe_3_O_4_@mSiO_2_@Au NPs is 1 nm, the electric field enhancement factors (|E/E_0_|^2^) are 11.11 and 9.20. It is known that the excitation rate of fluorophores in PEF phenomenon is directly proportional to the localized electric field intensity: the enhanced field strength correlates with increased photon absorption probability, consequently boosting fluorophore excitation rate. On the other hand, when fluorophores are close to metal NPs, the metal NPs can modify the local density of optical states (LDOS) around the fluorophores, increasing their radiative decay rate [40].

To verify an existence of PEF effect in the MF-induced aggregates of Fe_3_O_4_@mSiO_2_@Au-IR775 NPs, we explored changes in fluorescence lifetime of Fe_3_O_4_@mSiO_2_@Au-IR775 NPs aqueous dispersion with applied MF and without it using fluorescence lifetime imaging microscopy (FLIM) [41]. Figure 3(d, e) show the average fluorescence lifetime images and histograms for Fe_3_O_4_@mSiO_2_@Au-IR775 aqueous dispersion without and with MF application. The average fluorescence lifetime (τ_m_) for Fe_3_O_4_@mSiO_2_@Au-IR775 NPs acquired from different locations of the Petri dish with NPs dispersion in the absence of MF was measured to be 0.68 ns, 0.66 ns, 0.69 ns for locations L1, L2, L3, respectively. In contrast, τ_m_ was found to be significantly shorter (0.56 ns, 0.58 ns, 0.49 ns, and 0.46 ns) when an external MF was applied, with a clear correlation between areas with shorter lifetimes in FLIM images and higher fluorescence signal in the fluorescence images (Fig. 3(d, e)). Hence, the obtained results suggest that a plasmon coupling effect produced by the Au NPs becoming adjacent due to an MF-induced formation of Fe_3_O_4_@mSiO_2_@Au aggregates/clusters [21], resulting in a powerful PEF effect and leading to a significant shortening of the fluorescence lifetime and the amplification of fluorescence intensity [42, 43]. Interestingly, the FLIM of some areas, where Fe_3_O_4_@mSiO_2_@Au-IR775 aggregates were formed under an external applied MF (“e.g., “corners” C3 and C4 in Fig. 3(d, e)), did not show a notable reduction of the average fluorescence lifetime (approximately 0.65 ns and 0.63 ns, respectively), while the fluorescence signal at these locations is noticeably increased compared to that at the locations L1, L2, L3, where MF was not applied. It means that PEF effect in the areas C3 and C4 was not as pronounced and the observed fluorescence enhancement was mainly associated with the increased concentration of IR775 fluorophores. Correspondingly, it is naturally to suggest that the magnitude of the PEF effect is proportional to the MF strength at the selected locations and correlates with the fluorescence lifetime change.

At the same time, we hypothesize that there is a MF strength threshold range, above which PEF effect for Fe_3_O_4_@mSiO_2_@Au-IR775 NPs becomes substantial. For fluorescent molecules (IR775-silane) far away from metal nanoparticles (Au satellites), the fluorescence quantum yield *Q*_*0*_ and lifetime τ_0_ can be expressed as follows [44, 45]:

1 $${Q_0}=\frac{\Gamma }{{\Gamma +{K_{{\text{nr}}}}}}$$

2 $${\tau _0}=\frac{1}{{\Gamma +{K_{{\text{nr}}}}}}$$

Where Г is radiation decay rate and K_nr_ is nonradiation decay rate. When IR775 fluorophores are in the vicinity of Au satellites, their radiation decay increases with the generation of an extra radiative rate (Γ_m_) caused the Au NPs. In this case, the quantum yield *Q*_m_ and lifetime τ_m_ can be expressed as [46, 47]

3 $${Q_{\text{m}}}=\frac{{\Gamma +{\Gamma _{\text{m}}}}}{{\Gamma +{\Gamma _{\text{m}}}+{K_{{\text{nr}}}}}}$$

4 $${\tau _{\text{m}}}=\frac{1}{{\Gamma +{\Gamma _{\text{m}}}+{K_{{\text{nr}}}}}}$$

where Г_m_ is plasmon-enhanced radiation decay rate. As seen in Eqs. (3) and (4), the quantum yield Q_m_ of IR775 increases with an increase in the value of Γ + Γ_m_ (at constant K_nr_), while the fluorescence lifetime decreases. Thus, when the distance between the Au NPs and the IR775 fluorophore is within an appropriate range, the excitation and emission efficiency of IR775 are greatly enhanced, resulting in a significant enhancement of the fluorescence intensity [43]. It is worth also noting that the photostability of IR775-fluorophores should also increase in this case [12, 48, 49].

**Fig. 4:**
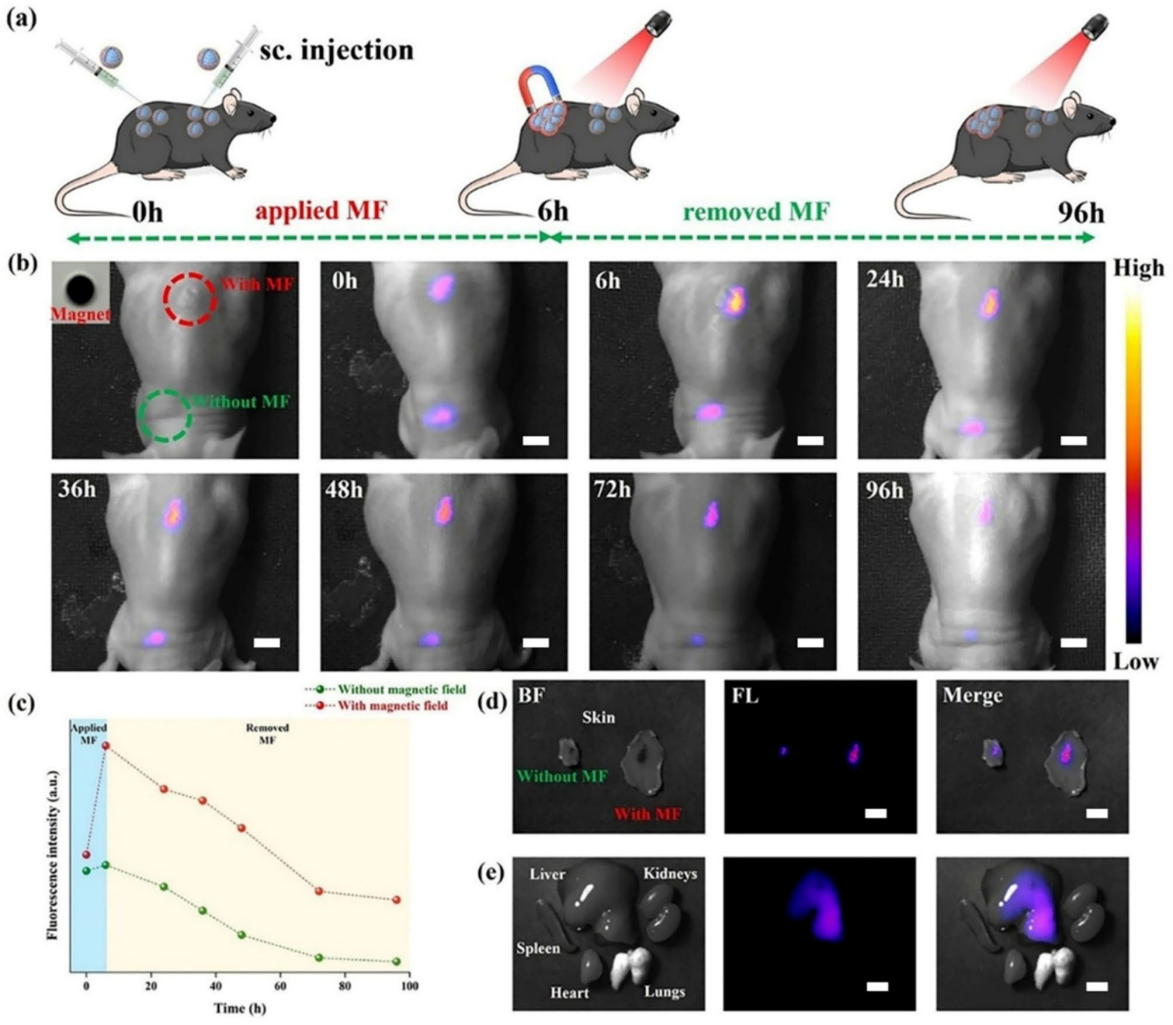
(**a**) Schematic illustration of the in vivo MF-induced PEF effect revealed by NIR fluorescence imaging. (**b**) NIR fluorescence images of the mouse at different time points after subcutaneous injection of Fe_3_O_4_@mSiO_2_@Au-IR775 NPs in different regions on the back of the mouse, with and without applied MF. (**c**) Fluorescence intensity versus time with and without applied MF regions in mouse. (**d**, **e**) Ex vivo NIR fluorescence imaging of main organs (heart, liver, spleen, lungs, kidneys, and skin) collected from mouse sacrificed 96 h after subcutaneous injection of Fe_3_O_4_@mSiO_2_@Au-IR775 NPs. Scale bar is 5 mm

At the next stage of the study, a possibility to obtain a MF-induced PEF effect with Fe_3_O_4_@mSiO_2_@Au-IR775 NPs in vivo was assessed in small animals (mice). First, the aqueous dispersion of Fe_3_O_4_@mSiO_2_@Au-IR775 NPs were twice subcutaneously (s.c.) injected in two different places on the back of one mouse. Next, a small round magnet NdFeB magnet was fixed at one of the locations for 6 h and then removed. NIR fluorescence imaging was performed at certain intervals after injection (0, 6, 24, 36, 48, 72, and 96 h), as illustrated in Fig. 4(a, b). Figure 4(b, c) shows that a significant enhancement of the fluorescence intensity was observed in the injection region after 6 h of MF application, while only very weak enhancement was seen in the location where the magnet was not applied (probably after excessive water was drained off the injection site). Along with this, the fluorescence area decreased, suggesting gathering of NPs by the applied magnet. The quantification of the fluorescence signal in the images is shown in Fig. 4(c), revealing about 2.1-fold more intense fluorescence after 6 h of MF application in comparison with that in absence of the external MF. Furthermore, as seen in Fig. 4(c), the fluorescence signal from the injected and MF-treated Fe_3_O_4_@mSiO_2_@Au-IR775 NPs exhibited a prolonged retention in the injection site: 96 h post-injection (and 90 h after the magnet was removed) NIR fluorescence from MF-treated subcutaneously injected NPs was ~ 6.8-fold more intense than that from the subcutaneously injected and MF-untreated NPs. This is apparently associated with the MF-induced formation of Fe_3_O_4_@mSiO_2_@Au-IR775 aggregates, which cannot be cleared from the body as fast as the non-aggregated NPs. After acquisition of the in vivo imaging 96 h post-injection of Fe_3_O_4_@mSiO_2_@Au-IR775 NPs, the mouse was sacrificed and its organs (skin, liver, kidneys, lungs, heart, spleen) were resected and imaged immediately. As demonstrated in Fig. 4(d), the images of the mouse skin samples harvested from two injection sites (with and without applied MF) also confirm an enhanced retaining of Fe_3_O_4_@mSiO_2_@Au-IR775 NPs at the site where MF was applied. Figure 4(e) shows the ex vivo images of the resected organs of mouse injected with Fe_3_O_4_@mSiO_2_@Au-IR775 NPs, revealing that the fluorescence signal is seen from liver and undistinguishable from kidney, spleen, lungs, and heart. It is known that subcutaneously injected NPs are drained into the lymphatic system, reach lymph nodes and, subsequently, enter the bloodstream. While smaller NPs (typically less than 10 nm) can be filtered by kidneys and excreted in urine, larger NPs are captured by liver, processed and excreted into bile, followed by excretion from the body with feces [50, 51]. An enhanced fluorescence signal from the liver is clearly associated with the hepatobiliary elimination pathway and it can be mainly associated with the non-aggregated NPs, while the NPs in MF-induced aggregates mainly retain at the injection site.

**Fig. 5:**
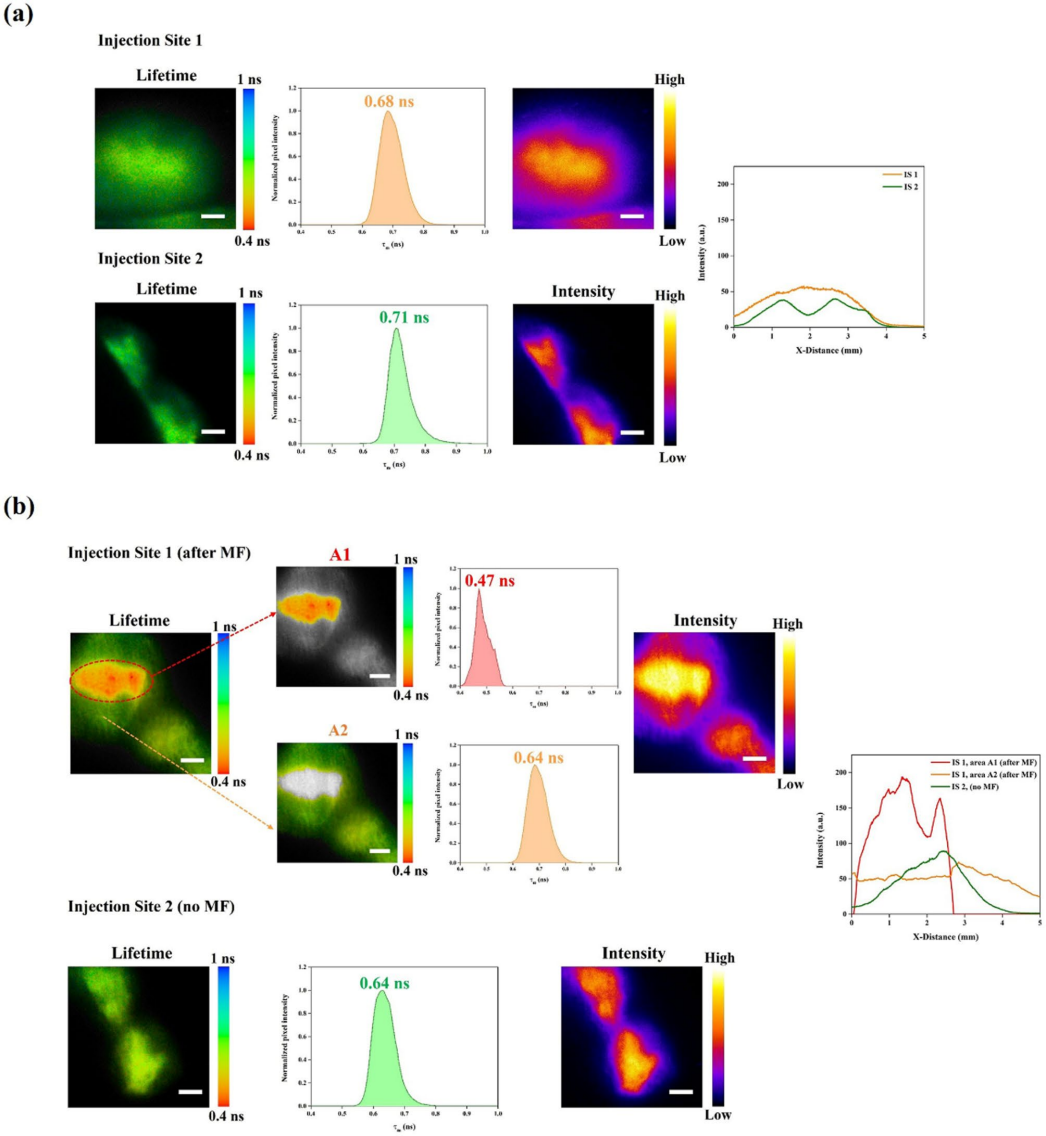
In vivo NiR fluorescence Lifetime imaging of mice acquired at 0 h (**a**) and 6 h (**b**) post subcutaneous injection of Fe_3_O_4_@mSiO_2_@Au-IR775 NPs, comparing regions with and without MF application, and corresponding fluorescence intensity profile before and after applied MF of Figure (a, b). Scale bar is 1 mm

To confirm that the observed increase in the fluorescence intensity 6 h post-injection is associated with MF-induced PEF effect, FLIM of the injection sites was performed in vivo in another s.c. injected mouse at 0 h and 6 h after injection, accessing the changes in fluorescence lifetimes for Fe_3_O_4_@mSiO_2_@Au-IR775 NPs before and after MF application (Fig. 5(a, b)). No noteworthy difference between fluorescence lifetimes at two injection sites (IS 1 and IS 2) was found before MF application (the average fluorescence Lifetimes at IS 1 and IS 2 were found to be 0.68 ns and 0.71 ns, respectively). Similarly, no notable difference in the fluorescence intensity was observed. Six hours after injection, the fluorescence lifetime of the Fe_3_O_4_@mSiO_2_@Au-IR775 NPs at IS 2 (where the MF was not applied did not change much (from 0.71 ns to 0.64 ns). In contrast the FLIM image of IS 1 reveal two clearly distinct areas. While the average fluorescence Lifetime in area 1 (A1) was found to be 0.47 ns (changing from 0.68 ns at 0 h time point, apparently as a result of MF application), the average fluorescence Lifetime in area 2 (A2) was ~ 0.64 ns, which is almost the same as in IS 1 at 0 h time point and in IS 2, where MF was not applied. Moreover, fluorescence intensity in A1 is also drastically higher than in A2. We believe that the obtained data clearly prove the PEF effect occurrence in A1, while it is not revealed at A2. One can suggest that the difference between A1 and A2 is in MF strength; this difference is similar to that shown in Fig. 3(d, e) (C1 and C2 vs. C3 and C4). Overall. the obtained FLIM images allow us to solidify our hypothesis that not only shortening of the fluorescence lifetime correlates with the MF strength, but there is a MF strength threshold range, below which PEF effect for Fe_3_O_4_@mSiO_2_@Au-IR775 NPs is insignificant. It should be also noted that the lifetime changes correlate with the changes in fluorescence intensity, similarly as in the batch experiment (Fig. 3) the shorted lifetime, the more intense fluorescence is. Hence, the FLIM imaging results presented in Fig. 5 confirmed that a significant localized PEF effect was induced in vivo by the application of external MF to the s.c. injected Fe_3_O_4_@mSiO_2_@Au-IR775 NPs.

**Fig. 6:**
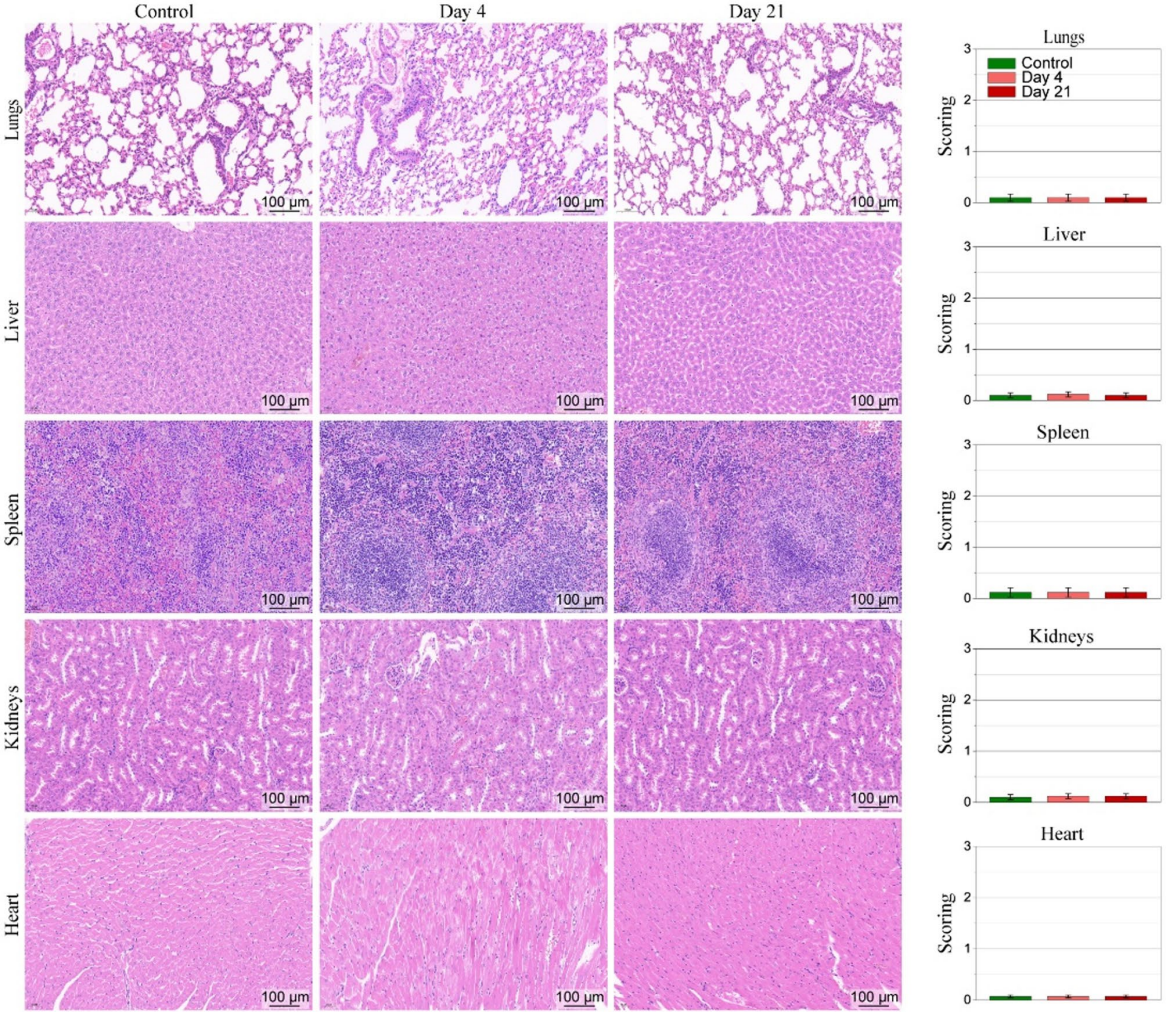
Representative images of histopathological analysis show the lungs, liver, spleen, kidneys, and heart sections stained with H&E at different treatment groups (control (healthy mouse without injection), mouse 4 days after s.c. injection of Fe_3_O_4_@mSiO_2_@Au-IR775 NPs and mouse 21 days after injection). Graphs show the histopathology scoring of lungs, liver, spleen, kidneys, and heart tissue in different groups. Data are presented as mean ± SD

Finally, to assess potential toxicity of Fe_3_O_4_@mSiO_2_@Au-IR775 NPs in in vivo applications, the s.c. injected mice were sacrificed at different time points (4 and 21 days) after injection, their vital organs (lungs, liver, spleen, kidneys, and heart) were collected and histological analysis was performed [52–54]. Figure 6 shows representative images of histopathological analysis of lungs, liver, spleen, kidneys, and heart sections stained with H&E at different treatment groups. The results of the histological analysis revealed the injected dosage of the Fe_3_O_4_@mSiO_2_@Au-IR775 NPs (200 µL, 1 mg/mL) did not cause any detectable toxicity in the s.c. injected mice, suggesting good biocompatibility safety of the Fe_3_O_4_@mSiO_2_@Au-IR775 nanoformulation in in vivo applications.

## Conclusions

This study reports a phenomenon of magnetic field-induced plasmon-enhanced fluorescence using Fe_3_O_4_@mSiO_2_@Au-IR775 core@shell@satellite magnetoplasmonic nanoparticles and its application for NIR fluorescence bioimaging. The synthesized NPs demonstrate magnetophoretic ability and also form aggregates under external MF. The MF-induced aggregation may lead to plasmon coupling between Au satellites of different NPs, enhancing local electric field strength and consequently amplifying the fluorescence of IR775 fluorophores in the shell of Fe_3_O_4_@mSiO_2_@Au-IR775 NPs. This localized enhancement of fluorescence intensity correlates with localized shortening of the fluorescence lifetime, confirming PEF effect induced by MF application to NPs. The application of external MF to the NPs subcutaneously injected into mice lead to the significant enhancements of NIR fluorescence intensity (~ 2.1-fold after 6 h of MF application) in comparison with that from the injected NPs in absence of MF. Besides, fluorescence from the subcutaneously injected and MF-treated NPs at 90 h after MF application (and 96 h post injection) was ~ 6.8-fold more intense than that from the subcutaneously injected and MF-untreated NPs, suggesting at MF-induced NPs aggregates cannot be cleared from the body as fast as the NPs non-treated with MF. Importantly, the FLIM imaging in vivo revealed that fluorescence lifetime from the part of injected and MF-treated NPs was significantly shortened (from ~ 0.68 ns to ~ 0.47 ns) after MF application, along with a significant increase in fluorescence intensity. At the same time, the average Lifetime from another part of MF-treated NPs injection site was found to be the same as in the MF-untreated injection site 6 h after injection (0.64 ns). The obtained FLIM imaging results allowed us to suggest that the fluorescence lifetime shortening correlates with the MF strength and there is a threshold for MF strength, below which PEF effect for Fe_3_O_4_@mSiO_2_@Au-IR775 NPs is insignificant. Histological studies of the main mouse organs showed no detectable toxicity for treated mice, suggesting good biocompatibility of the Fe_3_O_4_@mSiO_2_@Au-IR775 nanoformulation. Thus, it was demonstrated that the MF-induced PEF effect in a magnetoplasmonic nanoplatform can be employed for targeted NIR fluorescence bioimaging. It should be noted that that while a subcutaneous injection has been employed in this work, the studies are on the way to co-load molecular drugs (MD) into Fe_3_O_4_@mSiO_2_@Au-IR775 NPs and enable MF-induced, PEF imaging-guided synergistic cancer therapy from i.v. injected Fe_3_O_4_@mSiO_2_-MD@Au-IR775 NPs. A possibility to control PEF by the external MF with variable strength may be of interest for other imaging and sensing applications.

## Supplementary Information

Supplementary Material 1

## Data Availability

No datasets were generated or analysed during the current study.
