# Supplementary material for: Magnetic field-induced plasmonic enhancement of near infrared fluorescence from a Magnetoplasmonic nanoplatform for bioimaging applications
Source: J Nanobiotechnology. 2025 Sep 29;23:616. doi: 10.1186/s12951-025-03691-6 (PMC12482384; doi:10.1186/s12951-025-03691-6)
Supplement: Supplementary file 1 — Supplementary Material 1 [file 12951_2025_3691_MOESM1_ESM.docx]

**Magnetic field-induced plasmonic enhancement of near infrared fluorescence from a magnetoplasmonic nanoplatform for bioimaging applications**

Siqi Gao^†^, Jiantao Liu^†^, Iuliia Golovynska, Zhenlong Huang, Yiqiang Wang, Hao Xie, Rana Zaki Abdul Bari, Hao Xu, Junle Qu, Tymish Y. Ohulchanskyy

**Corresponding author. Email:* [*tyo@szu.edu.cn*](mailto:tyo@szu.edu.cn)

^†^*Contributed equally to this work.*

Key Laboratory of Optoelectronic Devices and Systems of Ministry of Education and Guangdong Province, College of Physics and Optoelectronic Engineering, Shenzhen University, Shenzhen 518060, China


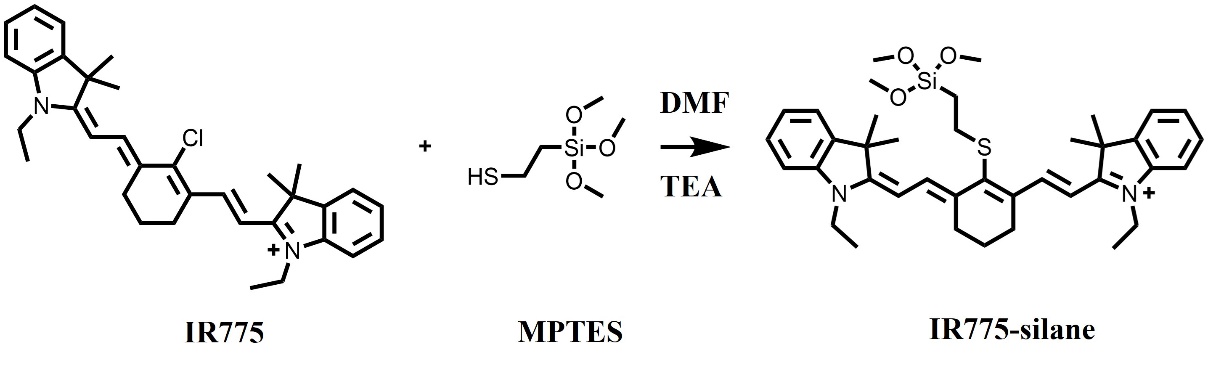


**Figure S1** Synthesis of MPTES-conjugated IR775.


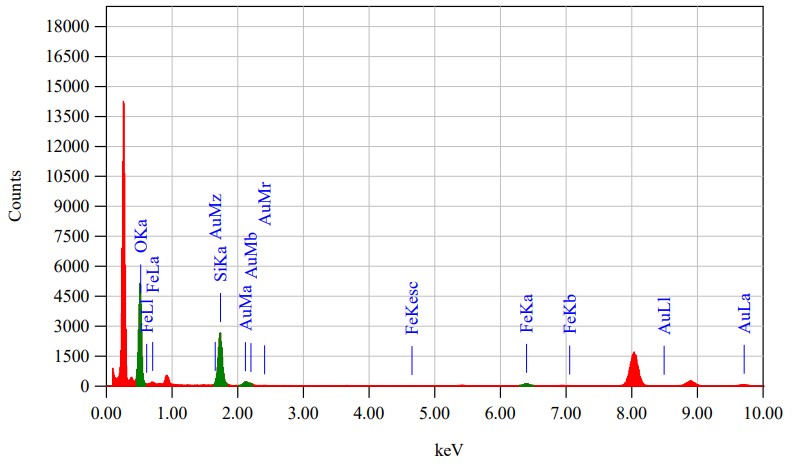


**Figure S2** Energy-dispersive X-ray (EDX) spectrum of Fe_3_O_4_@mSiO_2_@Au-IR775 NPs.

**Table S1 Quantitative Analysis**

| **Element** | **(kev)** | **Mass%** |
| --- | --- | --- |
| O K | 0.525 | 53.29 |
| Si K | 1.739 | 36.61 |
| Fe K | 6.398 | 2.38 |
| Au M | 2.120 | 7.71 |
| Total | / | 100 |


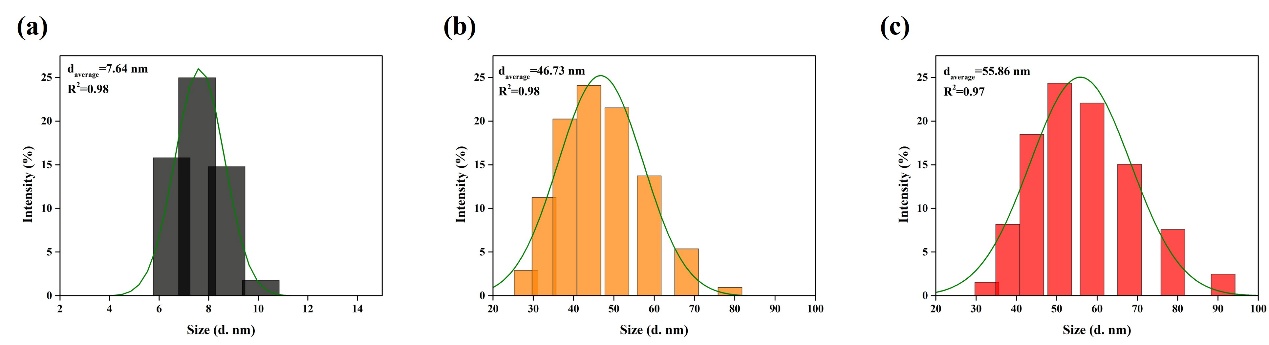


**Figure S3** Size distribution for Fe_3_O_4_ (a), Fe_3_O_4_@mSiO_2_ (b) and Fe_3_O_4_@mSiO_2_@Au-IR775 (c) NPs obtained using DLS measurement.

**Table S2** TEM and DLS results for NPs size

|  | Fe_3_O_4_ | Fe_3_O_4_@mSiO_2_ | Fe_3_O_4_@mSiO_2_@Au-IR775 |
| --- | --- | --- | --- |
| TEM results | 8 nm | 44 nm | 51 nm |
| DLS results | 7.64 nm | 46.73 nm | 55.86 nm |


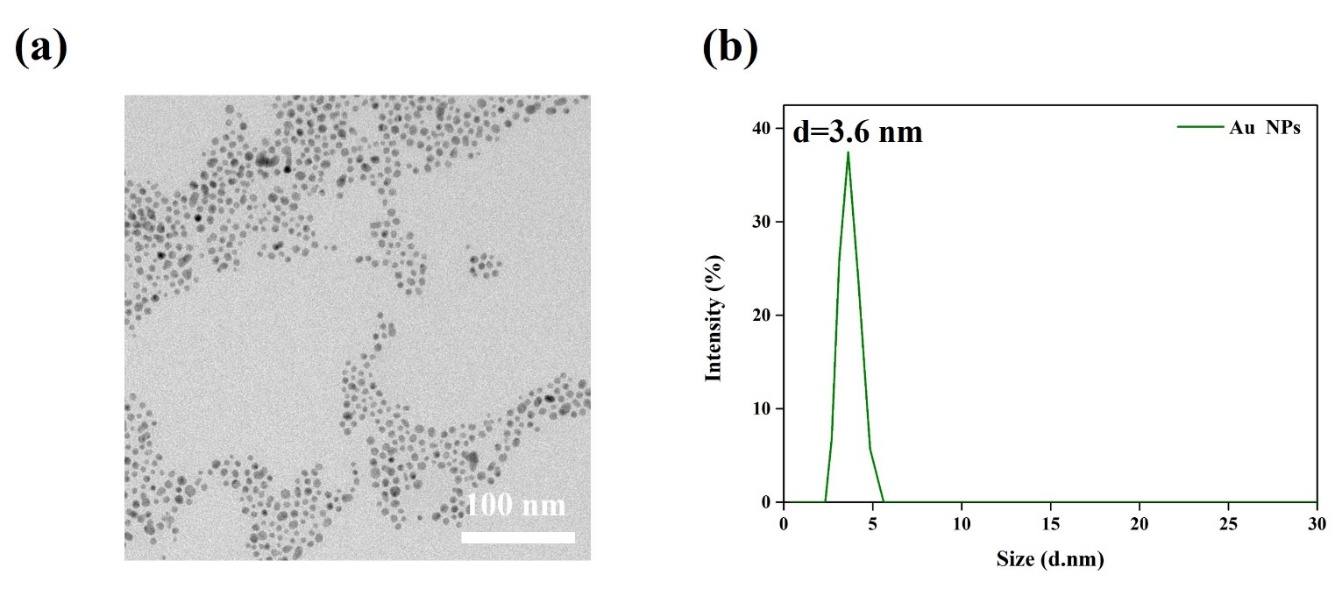


**Figure S4** TEM image of Au NPs (a), and their size distribution in water dispersion (DLS data) (b).


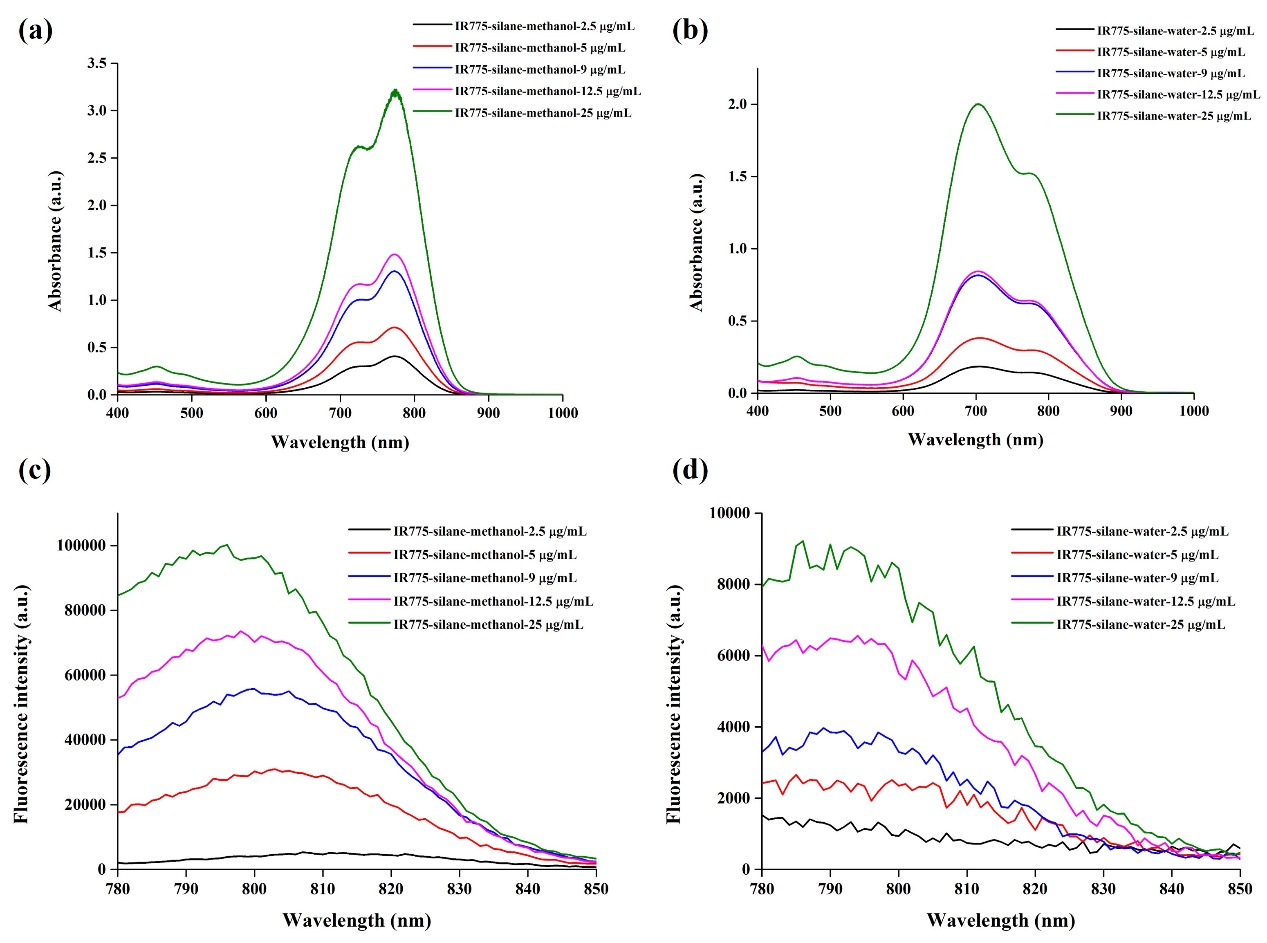


**Figure S5** Absorption (a, b) and fluorescence (c, d) spectra of IR775-silane in methanol and water.


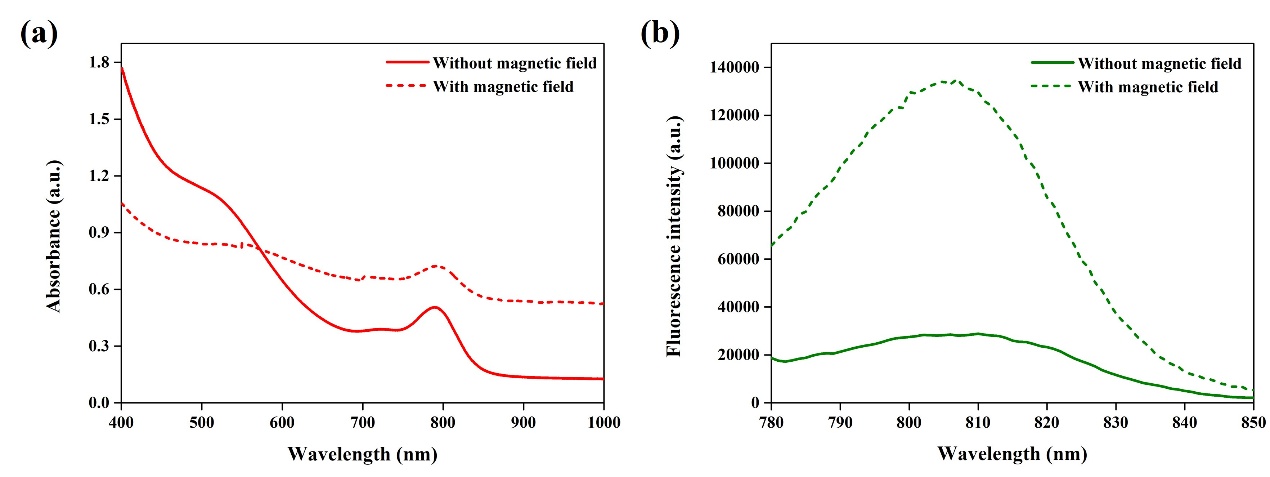


**Figure S6** Absorption spectra (a) and fluorescence spectra (b) of Fe_3_O_4_@mSiO_2_@Au-IR775 NPs dispersion with and without applied MF.


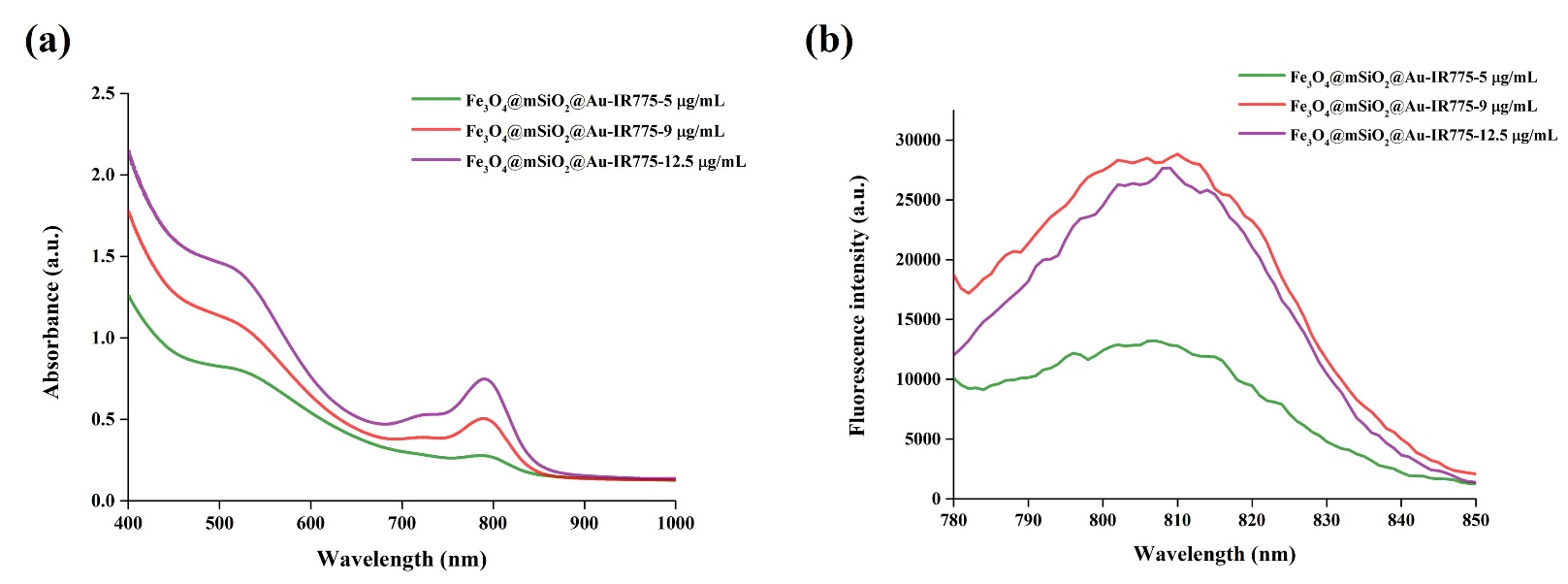


**Figure S7** Absorption (a) and fluorescence (b) spectra of Fe_3_O_4_@mSiO_2_@Au-IR775 with IR775-silane added at different concentrations.


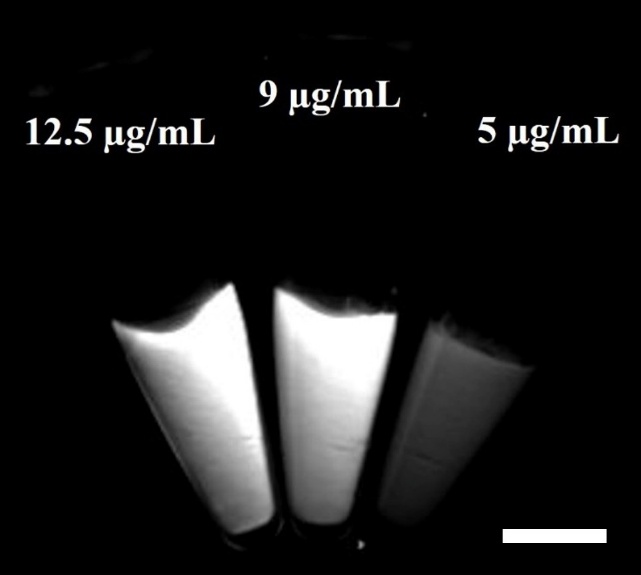


**Figure S8** NIR fluorescence image of Eppendorf tubes with Fe_3_O_4_@mSiO_2_@Au-IR775 dispersion with IR775-silane added at different concentrations. The image was acquired using an NIR camera, an 850 nm long-pass filter and 808 nm laser excitation (power density=20 mW/cm^2^, exposure time=200 ms). Scale bar is 10 mm.


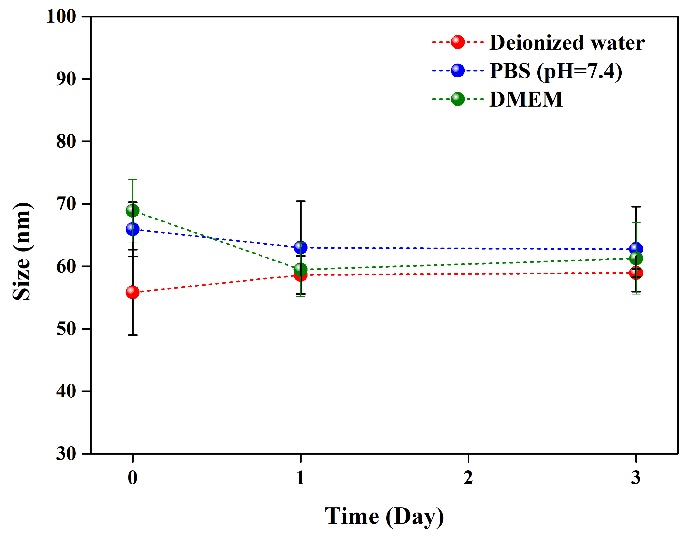


**Figure S9** DLS results (changes in hydrodynamic size in time) for Fe_3_O_4_@mSiO_2_@Au-IR775 NPs at deionized water, PBS (pH=7.4), and DMEM.


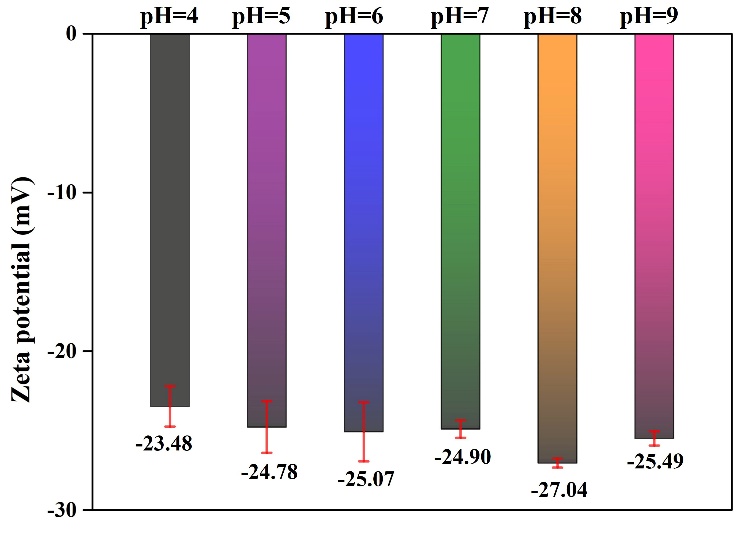


**Figure S10** Zeta potential of Fe_3_O_4_@mSiO_2_@Au-IR775 NPs at different pH.


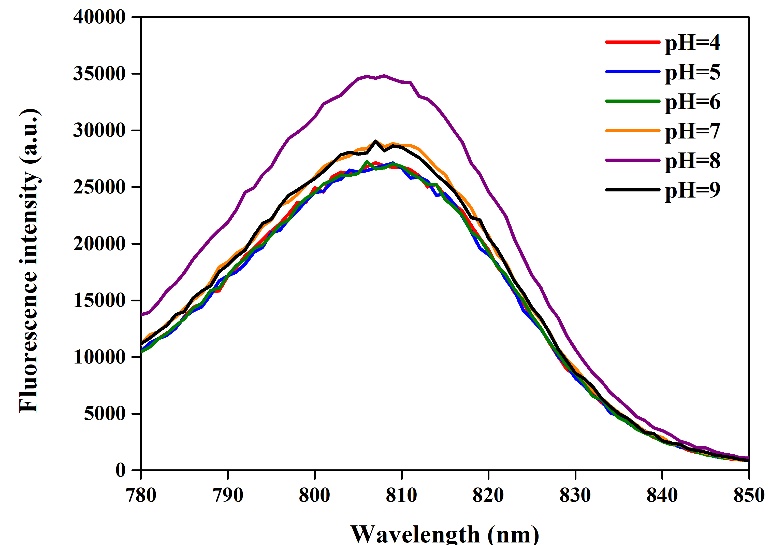


**Figure S11** Spectral of fluorescence intensity of Fe_3_O_4_@mSiO_2_@Au-IR775 suspension under different pH conditions.


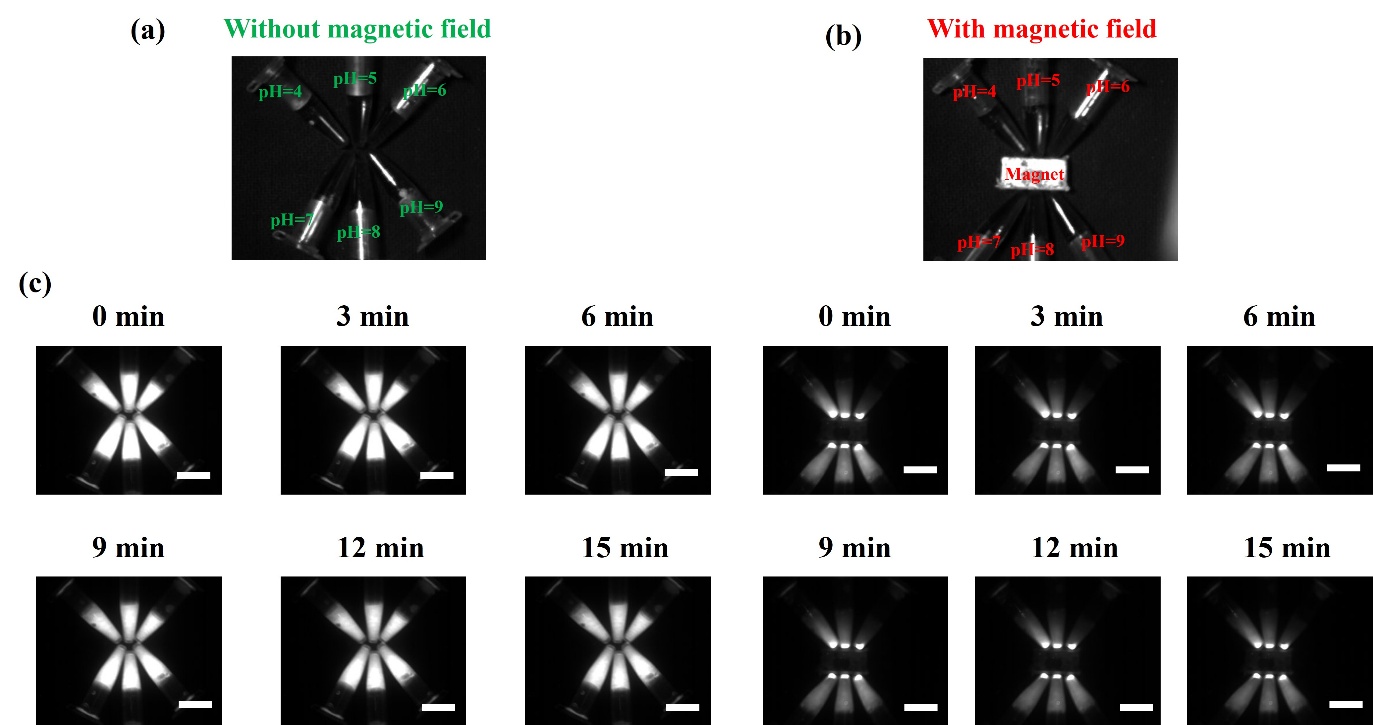


**Figure S12** (a, b): Bright field images of Eppendorf tubes with Fe_3_O_4_@mSiO_2_@Au-IR775 aqueous dispersions at different pH without (a) and with (b) applied MF. (c): corresponding NIR fluorescence images obtained by NIR camera (exposure time 200 ms) with 808 nm laser irradiation (power density=160 mW/cm^2^) for 0-15 min. Scale bar is 10 mm.


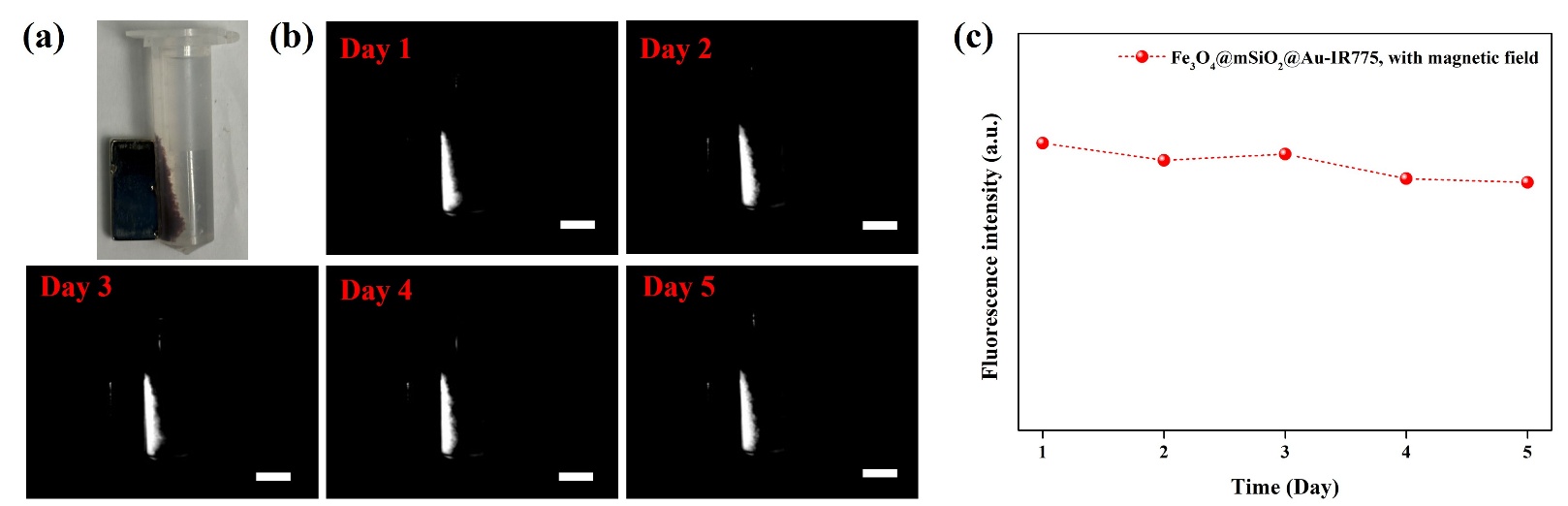


**Figure S13** (a) Photograph of Fe_3_O_4_@mSiO_2_@Au-IR775 (IR775 concentration=9 µg/mL, 2 mL) with the application of magnet, and (b) corresponding NIR fluorescence images at different time points (day1, day 2, day 3, day 4, and day 5) acquired using an NIR camera, an 850 nm long-pass filter and 808 nm laser excitation (power density=20 mW/cm^2^, exposure time=200 ms). (c) Dependence of fluorescence intensity of Fe_3_O_4_@mSiO_2_@Au-IR775 under applied MF on storage time. Scale bar is 10 mm.


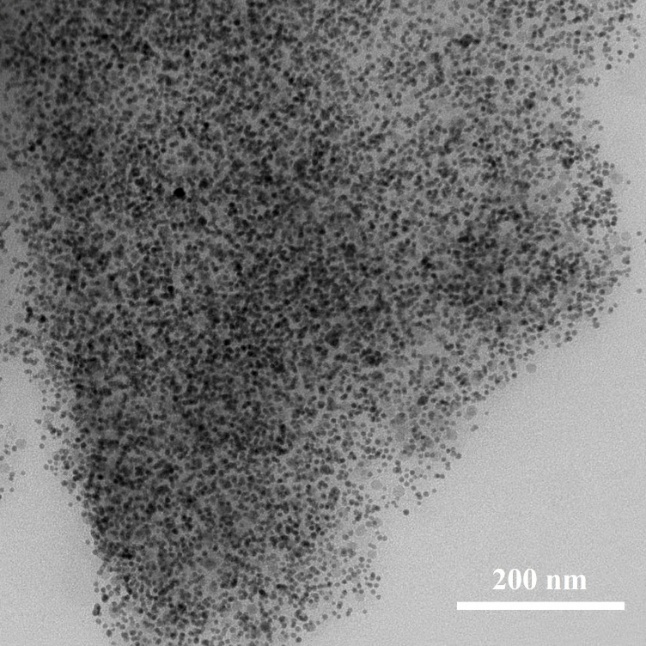


**Figure S14** TEM image of Fe_3_O_4_@mSiO_2_@Au-IR775 NPs aggregated after MF application.


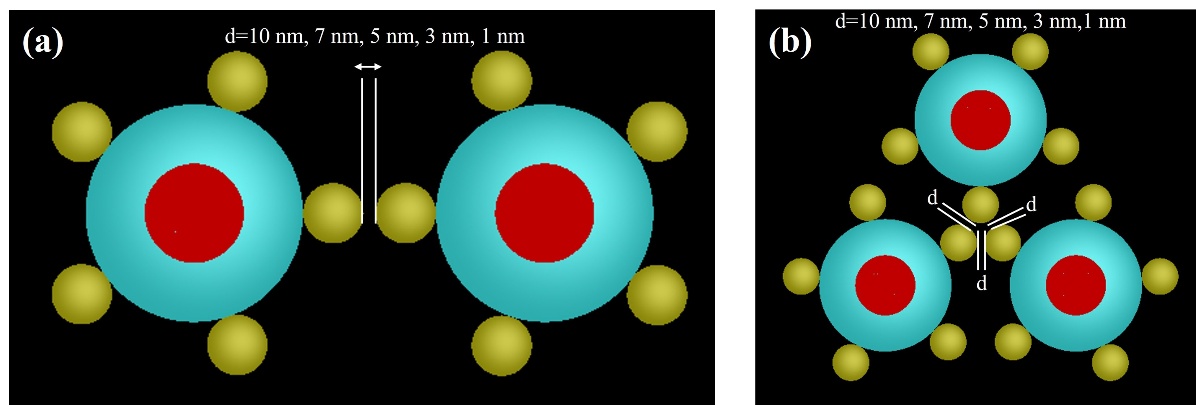


**Figure S15** Schematics of possible dimer (a) and trimer (b) structures of Fe_3_O_4_@mSiO_2_@Au NPs.


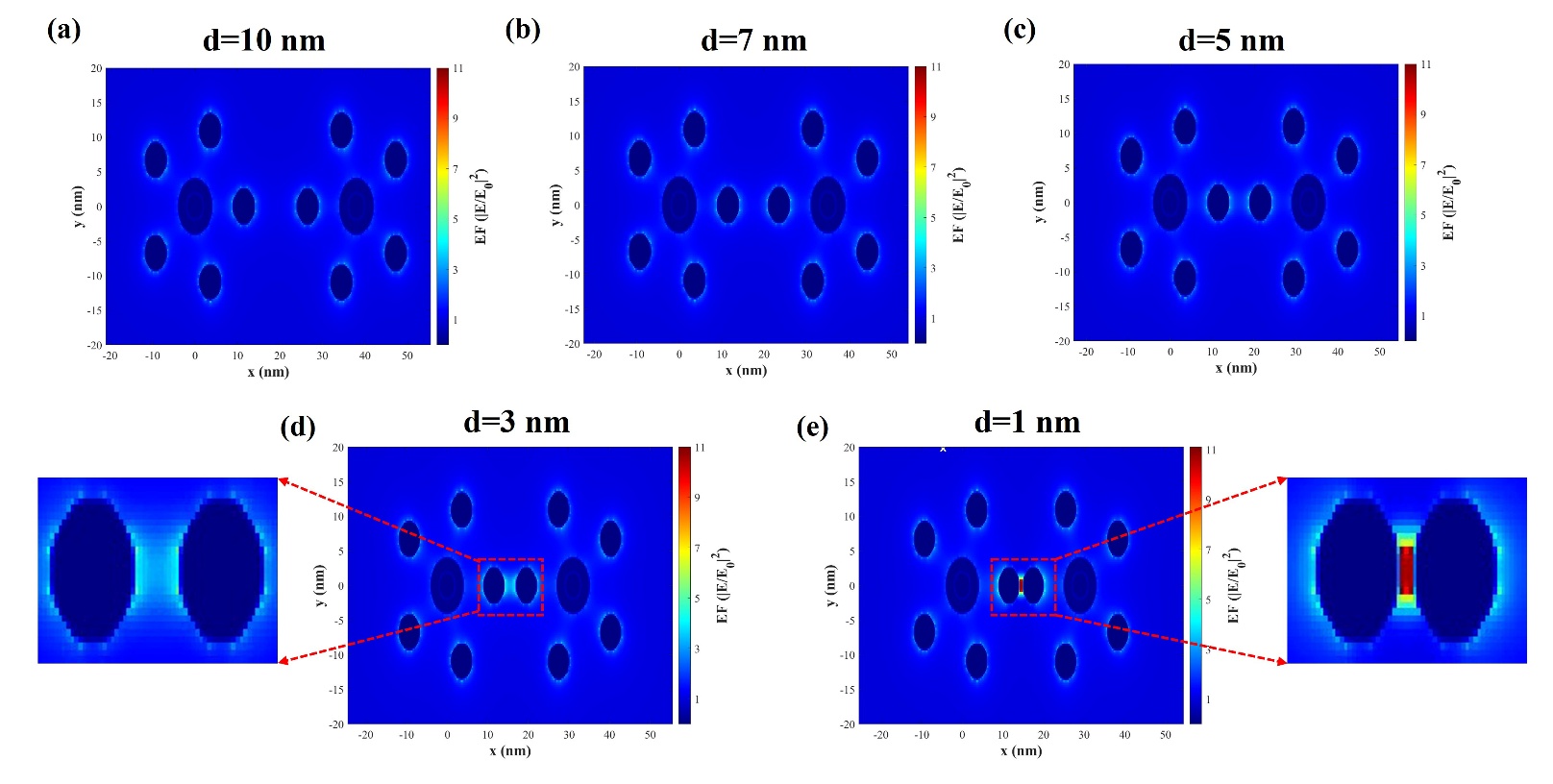


**Figure S16** FDTD simulated the electric field enhancement (|E/E_0_|^2^) distribution images for dimer structures of laser irradiated Fe_3_O_4_@mSiO_2_@Au NPs with shortening of distance between Au satellites.

**
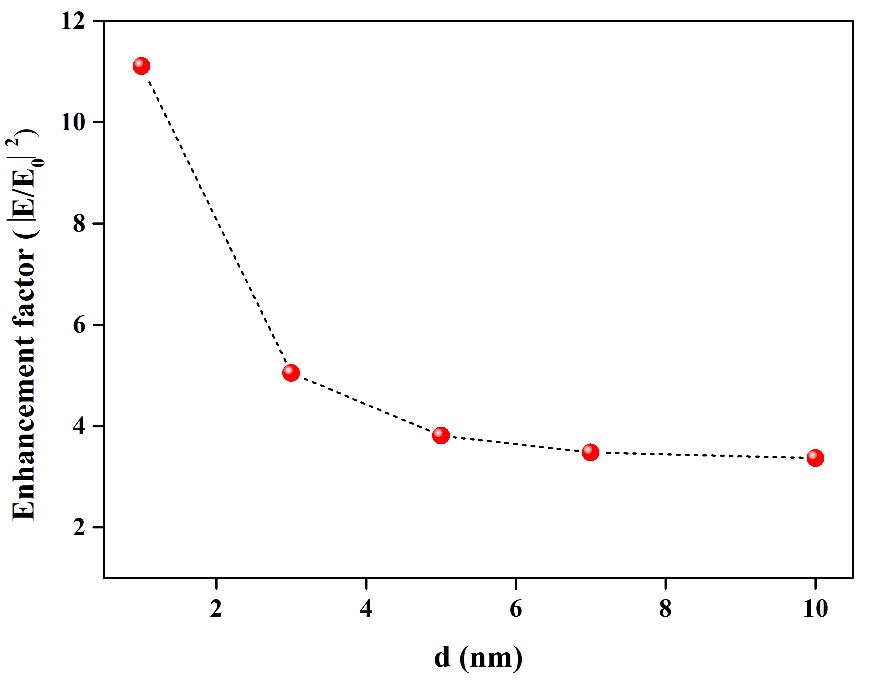
**

**Figure S1****7** Dependence of electric field enhancement factor (|E/E_0_|^2^) on distance between Au satellites in dimer of Fe_3_O_4_@mSiO_2_@Au NPs.


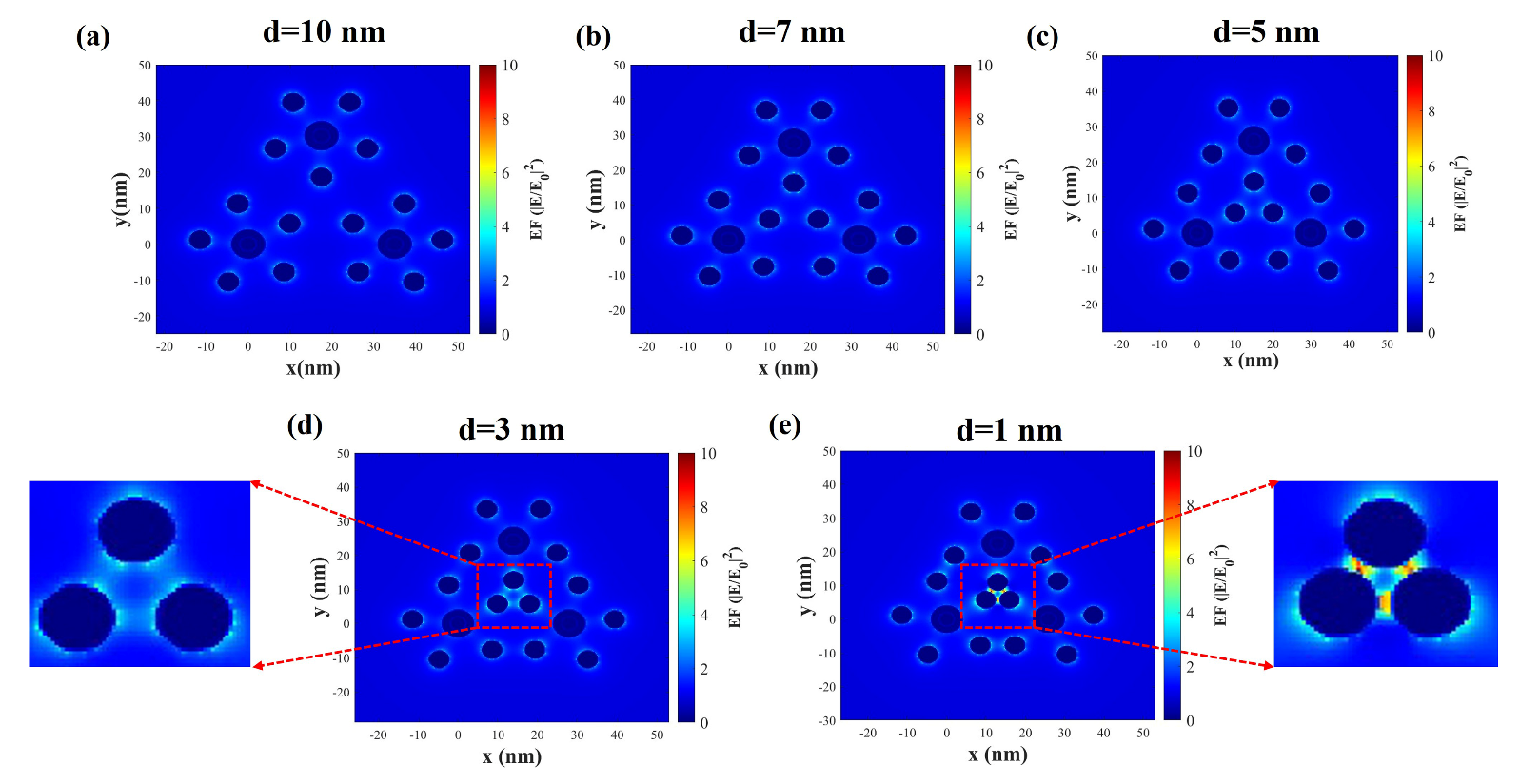


**Figure S1****8** FDTD simulated the electric field enhancement (|E/E_0_|^2^) distribution images for trimer structures of laser irradiated Fe_3_O_4_@mSiO_2_@Au NPs with shortening of distance between Au satellites.


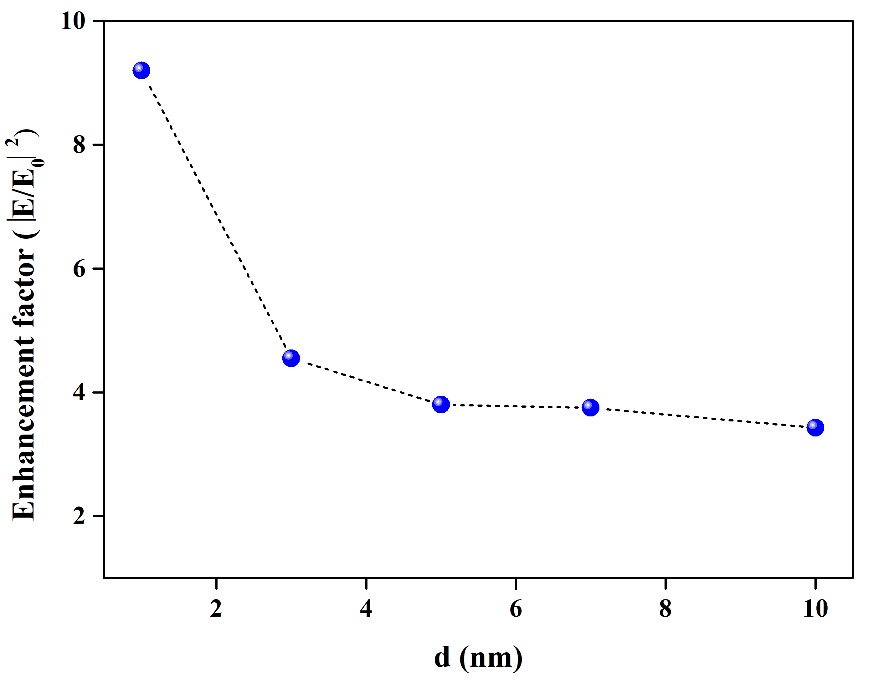


**Figure S19** Dependence of electric field enhancement factor (|E/E_0_|^2^) on distance between Au NPs for trimer structures of Fe_3_O_4_@mSiO_2_@Au NPs.
